# Hyperphosphorylated tau self-assembles into amorphous aggregates eliciting TLR4-dependent responses

**DOI:** 10.1038/s41467-022-30461-x

**Published:** 2022-05-16

**Authors:** Jonathan X. Meng, Yu Zhang, Dominik Saman, Arshad M. Haider, Suman De, Jason C. Sang, Karen Brown, Kun Jiang, Jane Humphrey, Linda Julian, Eric Hidari, Steven F. Lee, Gabriel Balmus, R. Andres Floto, Clare E. Bryant, Justin L. P. Benesch, Yu Ye, David Klenerman

**Affiliations:** 1 Department of Chemistry, University of Cambridge, Cambridge, UK; 2 UK Dementia Research Institute at Cambridge, Cambridge, UK; 3 Molecular Immunity Unit, Department of Medicine, MRC Laboratory of Molecular Biology, University of Cambridge, Cambridge, UK; 4 Cambridge Centre for AI in Medicine, University of Cambridge, Cambridge, UK; 5 Department of Chemistry, University of Oxford, Oxford, UK; 6 Department of Clinical Neurosciences, University of Cambridge, Cambridge, UK; 7 Department of Neuroscience Sheffield Institute for Translational Neuroscience, University of Sheffield, Sheffield, UK; 8 Cancer Research UK Cambridge Institute, University of Cambridge, Cambridge, UK; 9 Medicine and Veterinary Medicine, University of Cambridge, Cambridge, UK; 10 Department of Brain Sciences, Imperial College London, London, UK; 11 UK Dementia Research Institute at Imperial College London, London, UK

**Keywords:** Post-translational modifications, Mass spectrometry, Cellular imaging, Intrinsically disordered proteins, Protein aggregation

## Abstract

Soluble aggregates of the microtubule-associated protein tau have been challenging to assemble and characterize, despite their important role in the development of tauopathies. We found that sequential hyperphosphorylation by protein kinase A in conjugation with either glycogen synthase kinase 3β or stress activated protein kinase 4 enabled recombinant wild-type tau of isoform 0N4R to spontaneously polymerize into small amorphous aggregates in vitro. We employed tandem mass spectrometry to determine the phosphorylation sites, high-resolution native mass spectrometry to measure the degree of phosphorylation, and super-resolution microscopy and electron microscopy to characterize the morphology of aggregates formed. Functionally, compared with the unmodified aggregates, which require heparin induction to assemble, these self-assembled hyperphosphorylated tau aggregates more efficiently disrupt membrane bilayers and induce Toll-like receptor 4-dependent responses in human macrophages. Together, our results demonstrate that hyperphosphorylated tau aggregates are potentially damaging to cells, suggesting a mechanism for how hyperphosphorylation could drive neuroinflammation in tauopathies.

## Abstract (web summary)

In this work, the authors report that hyperphosphorylated recombinant tau spontaneously assembles into small, amorphous aggregates, which disrupt membranes and induce Toll-like receptor 4-dependent responses in human macrophages.

## Introduction

Intracellular neurofibrillary tangles (NFTs) constituting hyperphosphorylated tau proteins are a pathological hallmark of several neurodegenerative diseases including Alzheimer’s disease (AD)^1^, Pick’s disease, progressive supranuclear palsy, and corticobasal degeneration^2^, collectively called “tauopathies”. On the contrary, monomeric tau is highly soluble and intrinsically disordered, thereby showing little tendency in its native form for aggregation^3^. Thus, it is generally believed that tau proteins must undergo a sequence of biochemical and conformational changes before turning into misfolded substrates. Phosphorylation is the most common form of tau post-translational modifications found in vivo^4^ and has been suggested to be a pathological switch, leading to the formation of cytotoxic tau aggregates in NFTs^5^. On the other hand, when tau phosphorylation is inhibited, insoluble tau load and neurodegeneration are attenuated in vivo^6^, further implicating phosphorylation as a possible mechanism for the pathogenesis and progression of AD and other tauopathies.

**Fig. 1:**
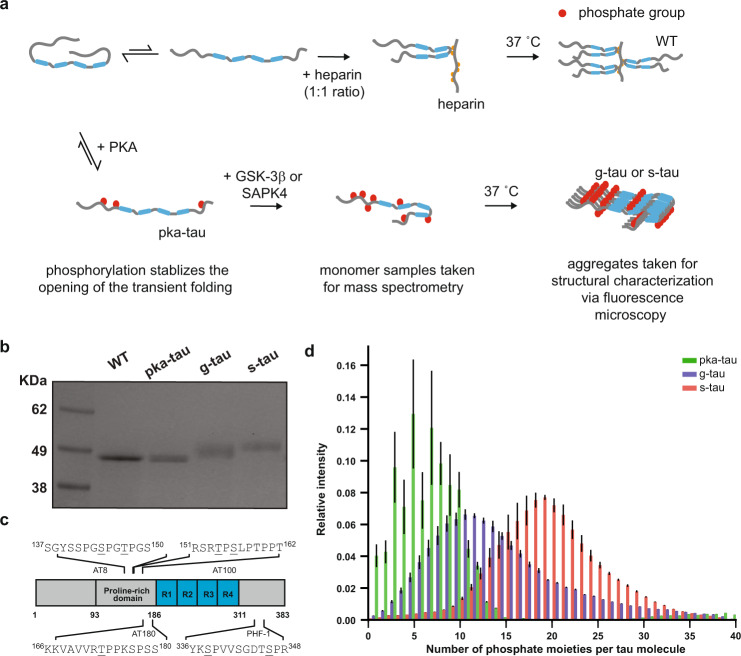
Sequential hyperphosphorylation of tau in vitro generates AD-specific epitopes. **a** A pictorial representation of the experimental design: WT tau was sequentially hyperphosphorylated, first by PKA and then by either GSK-3β or SAPK4 kinase. Hyperphosphorylation is shown to be able to cause an opening of such transient paperclip conformation^50^ and simultaneously stabilizes the α-helical structures, which is associated to the aggregation process^51^ Recent cryo-EM structures and computational study reveals heparin can stabilize the interaction between R2 and R3 (repeat domain 2 and 3) and hyperphosphorylation lowers the free-energy landscape of R3 and R4 for forming aggregation^15,55^. **b** Representative SDS-PAGE (4–12%, stained by Coomassie blue) across three independent experiments showed an upward shift of electrophoretic mobility for both g-tau and s-tau species, indicating successful phosphorylation reaction. **c** LC-MS/MS analysis revealed that both g-tau and s-tau tau were hyperphosphorylated at AD-specific epitopes, such as targeting sites of AT8, AT100, AT180, and PHF-1, highlighted along with the pictorial representation of WT tau. **d** High-resolution native mass spectrometry results indicated on average there were 7 phosphate groups per PKA-tau molecule, 11 phosphate groups per g-tau molecule and over 19 phosphate groups per s-tau molecule. Error bars represent ±s.d. from MCMC analysis.

However, the identification of phosphorylation sites responsible for neurotoxicity remains elusive, and the challenge mainly lies in the heterogeneous and combinatorial nature of tau phosphorylation. For instance, a 0N4R tau isoform harbors up to 71 potential phosphorylation sites, all of which can be modified by a multitude of kinases as well as phosphatases^7^. Most of these potential sites are located in the vicinity of the microtubule-binding domains (R1–4) in the proline-rich region and in the C-terminus of the molecule except for Ser^262^, Ser^293^, Ser^324^, and Ser^356^ (motif KXGS) in R1- 4 domains^8^ (Fig. 1b). Out of these potential phosphorylation sites about 30 sites have been reported to be aberrantly phosphorylated in the AD brain but not in the healthy control^9^ and commonly associated with tau aggregation processes such as incomplete binding and destabilization of microtubules, causing the transition from pre-tangles to NFTs^10–12^. Therefore, to examine the pathological consequences of tau (hyper)phosphorylation, we need to be able to reproducibly generate disease-relevant phospho-tau in quantities that meet the demand for subsequent structural and toxicity characterization studies.

An early study has demonstrated that prior phosphorylation with cAMP-dependent protein kinase A (PKA) can prime the tau proteins for other kinases by inducing conformational changes to allow further phosphorylation events^13^. Moreover, a recent study that screens for the effect of 352 human kinases has shown that glycogen synthase kinase 3β (GSK-3β) and stress-activated protein kinase 4 (SAPK4) were the most active protein kinases phosphorylating tau at AD-specific epitopes that were recognized by phospho-tau specific antibodies including AT8 (pSer^202^ and pThr^205^), AT180 (pThr^231^), AT100 (pThr^212^ and pSer^214^), and PHF-1 (pSer^396^ and pSer^404^)^10,14^ (the phospho-residues are numbered according to the longest 2N4R tau isoform). Thus, we used PKA followed by either GSK-3β or SAPK4 in a sequential manner to generate hyperphosphorylated tau with AD-specific epitopes in vitro. We validated the modifications on tau proteins using liquid chromatography-tandem mass spectrometry and high-resolution native mass spectrometry. Applying super-resolution microscopy and transmission electron microscopy, we investigated the effects of hyperphosphorylation on the aggregation process and morphology. Our results demonstrated that hyperphosphorylated tau could spontaneously assemble into small amorphous aggregates at physiological concentrations without the use of any external inducer. This is important since the use of external inducers like heparin, the canonical method to generate in vitro tau aggregates, have recently been cast into doubt when examination of recombinant and AD-derived aggregates of tau found differences in their conformations, properties, and activities^15,16^. We found that these hyperphosphorylated tau aggregates are more cytotoxic than the wild-type (WT) aggregates and can cause membrane destabilization and Toll-like receptor 4 (TLR4)-dependent responses in human macrophages. Our findings suggest that tau hyperphosphorylation produces cytotoxic aggregates through distinct mechanisms, offering potential explanations for how hyperphosphorylated tau contributes to the development and progression of AD and other tauopathies.

## Results

### AD-specific epitopes on in vitro hyperphosphorylated tau

Following a previously established protocol^10^, we performed in vitro phosphorylation assays, where recombinant WT tau of isoform 0N4R was sequentially phosphorylated by PKA and then either GSK-3β or SAPK4 (Fig. 1a). We examined the phosphorylation reactions by SDS-PAGE to confirm that the modified tau proteins had a characteristic upward shift when compared to their non-phosphorylated control (Fig. 1b). To characterize the phosphorylation sites, we digested phosphorylated tau monomers with trypsin and analyzed the corresponding tryptic peptides with liquid chromatography-tandem mass spectrometry (LC-MS/MS). Phosphorylation sites of PKA-phosphorylated tau (PKA-tau), GSK-3β phosphorylated tau (g-tau), and SAPK4 phosphorylated tau (s-tau) were mapped in greater detail in Supplementary Table 1. While both GSK-3β and SAPK4 are a priori serine/threonine kinases, some tyrosine residues were found to be phosphorylated in g-tau and s-tau, suggesting that prior phosphorylation by PKA may alter the recognition of tau by GSK-3β or SAPK4. Out of the 31 sites found to be phosphorylated in both g-tau and s-tau, our LC-MS/MS result revealed that both combinations of kinases afforded tau phosphorylation in AD-specific epitopes, including targeting sites of AT8, AT180, AT100, and PHF-1^17^ (Fig. 1c, Supplementary Fig. 1). By comparing with previous reports on AD-derived PHF tau^9^ and other (hyper)phosphorylated tau synthesized in vitro^13,18–20^, our g-tau and s-tau were the most disease-relevant species in terms of their phosphorylation sites (Supplementary Table 1).

To further assess the extent of phosphorylation, we employed high-resolution native mass spectrometry, which allowed us to determine the average number of phosphate groups per tau protein. The deconvoluted mass distribution for unmodified WT tau had its highest peak at 39.9 kDa, which corresponded to the full-length tau without the N-terminal methionine (Supplementary Fig. 2). The deconvoluted mass distribution for PKA-tau ranged between 40.0 and 41.0 kDa with the highest peak at 40.5 kDa, corresponding to 7 phosphate groups (80 Da each) per tau protein (Supplementary Fig. 2). The deconvoluted mass distribution for g-tau ranged between 40.0 and 42.0 kDa with the highest peak at 40.7 kDa, corresponding to 10 phosphate groups per tau protein (Supplementary Fig. 2). Furthermore, s-tau showed a mass distribution between 40.5 and 43.0 kDa with its highest peak at 41.4 kDa, equivalent to 19 phosphate groups per tau protein (Supplementary Fig. 2). Unlike tau characterized in control human brain tissues, which contains two to three phosphate groups, hyperphosphorylated tau in PHF derived from brain samples of AD patients harbor five to nine phosphate groups on average^21^. Therefore, both g-tau and s-tau can be considered hyperphosphorylated. We also found that PKA/SAPK4 had greater synergy than PKA/GSK-3β, not only in adding phosphate groups at more possible sites, as previously measured by our LC-MS/MS, but also in incorporating more phosphate moieties per tau molecule while all other reaction conditions being controlled (Fig. 1d). Such observation may be due to the potential crosstalk and regulation between kinases, more especially the potential inhibition of GSK-3β by PKA in this case as demonstrated previously^22^.

### Hyperphosphorylated tau self-assembled to unstructured and amorphous oligomers

**Fig. 2:**
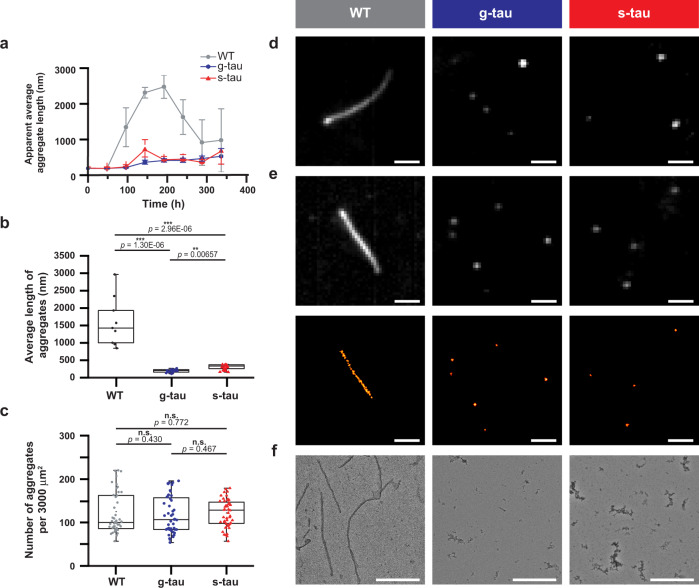
Hyperphosphorylation enables WT tau to self-polymerize into small amorphous aggregates without external inducers. **a** The apparent average length of tau aggregates, monitored by fluorescence microscopy, was analyzed, and plotted as a function of time (data are presented as mean values ± s.d. across three independent experiments for each tau species). **b** Summary plot based on our results from super-resolution microscopy indicated that at 96 h of incubation the average length of heparin-induced WT aggregates (1450 ± 700 nm) was significantly larger than both g-tau (190 ± 50 nm) and or s-tau (280 ± 90 nm) (*n* = 9 across three independent experiments for each tau species). **c** Summary plot based on our results from fluorescence microscopy indicated the number of aggregates was not significantly different among these three tau species (*n* = 48 across three independent experiments for each tau species). **d** Representative pFTAA diffraction-limited images of different tau species after 96 h of incubation: while WT tau under the induction of heparin grew into fibrillar aggregates, both g-tau and s-tau self-assembled into small, non-fibrillar aggregates which largely remained as diffraction-limited spots. Scale bar: 1.0 μm. **e** Representative ThX stacked and super-resolved images of different tau species after 96 h of incubation. Scale bar: 1.0 μm. **f** Morphological examination of all three tau species by TEM after 96 h of incubation. Scale bar: 500 nm. For **b** and **c**, boxplots represent median value and interquartile range (25–75% percentiles) excluding outliers. The *P*-values are based on unpaired two-sided Student’s *t*-test: ***p < 0.001; *p < 0.05; n.s. non-significant. For **d**–**f**, images were representative across three independent experiments for each tau species. Source data are provided as a Source Data file.

Several studies have characterized the self-assembly process of hyperphosphorylated tau in vitro, suggesting that soluble phosphorylated tau can undergo spontaneous liquid-liquid phase separation and ultimately form tau aggregates under cellular conditions^23,24^ Therefore, both g-tau and s-tau were compared side-by-side with heparin-induced WT tau in order to examine the effect of hyperphosphorylation on the aggregation kinetics at neuronal tau concentration (2 μM)^25^. The pentameric oligothiophene pFTAA with a high affinity to tau aggregates was employed to follow the evolution of aggregation in vitro^26^ (Supplementary Fig. 3a). Representative fluorescence images of tau aggregates at different time points are shown in Fig. 2d and Supplementary Fig. 3b. To obtain quantitative insights from our TIRF images, the apparent average length of pFTAA-positive aggregates was plotted as a function of time (Fig. 2a). The heparin-induced WT tau first elongated into fibrillar aggregates with an apparent average length of 2400 ± 500 nm and then slowly fragmented into smaller aggregates, in agreement with the aggregation-and-fragmentation pattern as previously observed^26^. On the other hand, g-tau and s-tau slowly polymerized into small aggregates with apparent average lengths of 530 ± 220 nm and 670 ± 400 nm, respectively. Fibrils above 1 μm were observed only rarely for s-tau. Critically, both these hyperphosphorylated tau species remained as small aggregates over 300 h of incubation. Hence, our results suggested that both hyperphosphorylated tau species could self-assemble to form pFTAA-active aggregates and that such aggregations of hyperphosphorylated tau were significantly slower than that of heparin-induced WT tau, consistent with previously reported study^23^.

To further characterize the structure of tau aggregates, hyperphosphorylated tau aggregates were then prepared for super-resolution microscopy using a recently developed thioflavin-T (ThT) derivative, called Thioflavin-X (ThX)^27^ (Supplementary Fig. 3a). The transient nature of ThX binding to the β-sheet motif and its low fluorescence quantum yield in solution creates the necessary blinking events which enable us to super-resolve the morphology of individual tau aggregates^27^. Using this approach, we achieved a mean localization precision of 18.9 nm and an image resolution of 29.4 nm, determined by Fourier ring correlation analysis (Supplementary Fig. 4e, f). The samples were imaged after 96 h of incubation, the earliest timepoint at which self-assembled hyperphosphorylated tau and heparin-induced WT tau appeared to have distinctive structures. Based on ThX-based fluorescence images, the average length for WT tau was 1450 ± 700 nm while g-tau and s-tau aggregates had average lengths of 190 ± 50 nm and 280 ± 90 nm, respectively (Fig. 2b). By comparison, the filament length of WT tau was significantly larger than those formed by g-tau and s-tau while the number of aggregates was comparable among different tau species at 96 h timepoint, further corroborating our pFTAA results above (Fig. 2c). The size distribution obtained from ThX fluorescence images further revealed morphological differences between WT tau and hyperphosphorylated tau: more than 25% of WT tau formed aggregates longer than 1000 nm, whereas no g-tau and only 1.2% of s-tau were over this size threshold. On the other hand, about 99% of g-tau and 89% of s-tau aggregates stayed below a length of 500 nm, while only 50% of WT tau aggregates were under that threshold (Supplementary Fig. 4d). Representative ThX images of tau aggregates are shown in Fig. 2e and Supplementary Fig. 4a–c.

As the integrated fluorescence intensity from probes such as pFTAA or ThX can be influenced by the structure of tau aggregates^26,27^, samples of hyperphosphorylated tau aggregates were further investigated by transmission electron microscopy (TEM). TEM results showed that only non-fibrillar, amorphous aggregates were detected for both hyperphosphorylated species and abundant, relatively short fibrillar aggregates, for WT tau (Fig. 2f), supporting our previous observations. Together, these results showed that hyperphosphorylation was sufficient to induce tau aggregation.

To estimate the extent of aggregation at 96 h timepoint, we evaluated the bands from SDS-PAGE by densitometry, since high-order tau aggregates are SDS-stable and thereby unable to enter the gel^28,29^. By running various concentrations of tau aggregates and the monomer, we ensured the selected concentrations were within the linear range of our densitometric detection. Then by comparing the band intensities between aggregates and monomer, our SDS-PAGE result demonstrated that about 2% of WT tau and 1% of g-tau and s-tau had formed SDS-stable aggregates (Supplementary Fig. 5a).

### Hyperphosphorylated tau aggregates effectively disrupt lipid membrane

**Fig. 3:**
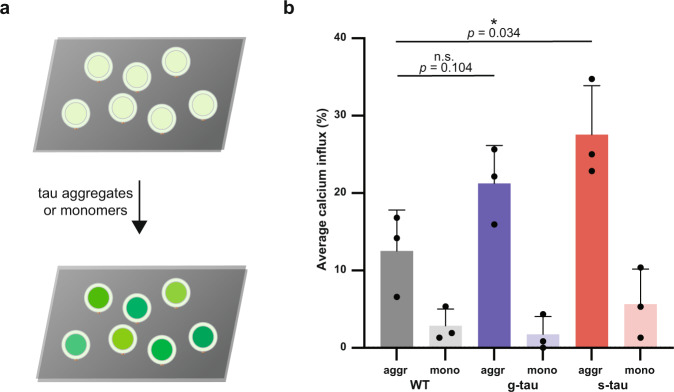
Hyperphosphorylated tau amorphous aggregates disrupt membrane integrity more effectively than WT fibrillar aggregates. **a** The ability to disrupt membrane integrity, an important aspect that explains the toxicity of protein aggregates, was quantified by calcium influx caused by each tau species. The percentage of Ca^2+^ influx was normalized by the maximum Ca^2+^ influx induced by ionomycin. **b** Our results from the liposomal assay revealed that WT tau aggregates caused 12 ± 2% calcium influx, g-tau aggregates elicited 21 ± 5% calcium influx, and s-tau aggregates induced 28 ± 6% calcium influx. All of the monomeric controls caused less calcium influx than their corresponding aggregate samples. Hence, s-tau aggregates can better permeabilize synthetic lipid bilayer than WT aggregates (data are presented as mean values ± s.d. of *n* = 3 for each tau species, the *P*-values are based on unpaired two-sided Student’s *t*-test: *p < 0.05, n.s. non-significant). Source data are provided as a Source Data file.

We next investigated whether such pronounced structural changes in tau aggregates might translate to a gain of toxic function for tau. To address this question, a high-throughput membrane destabilization assay was used to measure the ability of tau aggregates to disrupt lipid membrane integrity as a proxy of tau cytotoxicity^30^. This approach allowed us to differentiate and quantify the membrane disruptive capability of different protein aggregates, including Aβ^31^, α-synuclein^32^, tau^33^, as well as those in human cerebrospinal fluid from AD patients^34^ (Fig. 3a).

Heparin-induced WT tau aggregates and self-assembled hyperphosphorylated tau after 96 h of incubation along with respective monomer controls were applied at the same monomeric equivalent concentration (100 nM) to liposomal vesicles. The percentage of calcium influx was measured as an indication of membrane permeabilization capacity for different tau species. Our results showed s-tau aggregates induced 28 ± 6% calcium influx compared to ionomycin positive control, whereas WT tau aggregates caused 12 ± 2% calcium influx and g-tau aggregates elicited 21 ± 5% calcium influx in our liposomal assay (Fig. 3b). Therefore, s-tau aggregates were significantly more potent than both WT tau aggregates and s-tau monomer control (6 ± 5% of calcium influx) at permeabilizing membrane vesicles. On the other hand, g-tau aggregates did not elicit any significant membrane disruption compared to WT aggregates. Such functional differences between g- and s-tau might be due to their differential phosphorylation patterns and conformations.

### Hyperphosphorylated tau aggregates elicit inflammatory responses in human macrophages

In addition to intracellular hyperphosphorylated NFTs, neuroinflammation is also a prominent pathological feature of AD^35^. Immune cells from different origins—including brain-resident macrophages as well as bone-marrow-derived circulating monocytes—were recently observed to participate in the neurodegenerative process by secreting pro-inflammatory chemicals and cytokines which exert toxic effects on neurons^36^. Hence, neuroinflammation was further hypothesized to be a key pathological cause that accelerated the disease progression. In order to investigate the interplay between tau pathology and the innate immune responses and gain a better understanding of tau toxicity induced by hyperphosphorylation, we devised a macrophage cell assay by detecting intracellular calcium transients and production of reactive oxygen species (ROS) as immediate-early readouts of macrophage activation ^37^.

**Fig. 4:**
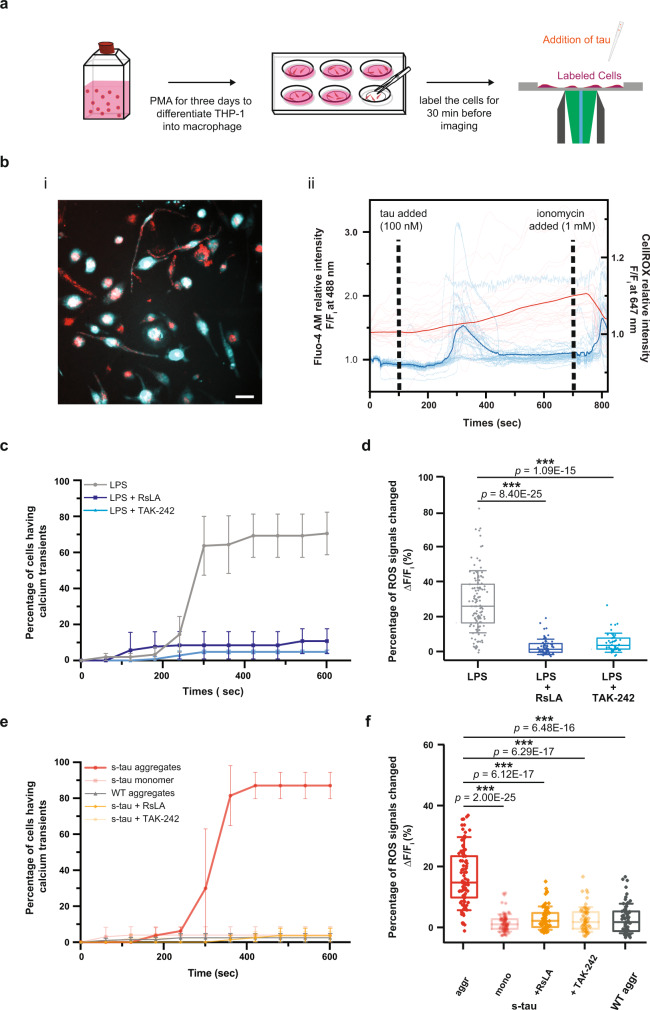
Hyperphosphorylated tau aggregates elicited calcium transient and stimulated ROS production in human macrophages in a TLR4-dependent manner. **a** A pictorial representation of the macrophage assay workflow. The cytosolic calcium and ROS level were simultaneously monitored as different tau aggregates were applied. **b** Representative image of dual-labeled macrophage (i) and calcium and ROS traces (ii) after the treatment of s-tau aggregates. Scale bar: 25.0 μm. **c**, **d** Summary plots of calcium transients (**c**) and ROX production (**d**) induced by LPS (*n* = 123). When treating the cells with RsLA (*n* = 70, F_(1,4)_ = 40.78) or TAK-242 (*n* = 47, F_(1,4)_ = 84.62), the LPS-induced calcium transients and ROS level elevation were significantly moderated. **e**, **f** Summary plots of calcium transients (**e**) and ROX production (**f**) induced by different tau species and treatment conditions. Compared to WT tau aggregates (*n* = 63), s-tau aggregates induced strong calcium transients and ROS production (*n* = 83, F_(1,4)_ = 174.88). Since s-tau monomer control did not elicit significant response (*n* = 89, F_(1,4)_ = 110.14), ATP and LPS contamination were controlled. The involvement of TLR4 was tested: by pre-treating the cells with RsLA (*n* = 65, F_(1,4)_ = 184.60) or TAK-242 (*n* = 62, F_(1,4)_ = 170.11), s-tau can no longer elicit significant response. For **c** and **e**, data are presented as mean values ± s.d. of three independent experiments. The *P*-values are based on two-way mixed ANOVA: all *p* < 0.001 except the one between LPS and RsLA which is *p* < 0.01. For **d** and **f**, boxplots represent median value and interquartile range (25–75% percentiles) excluding outliers. The *P*-values are based on unpaired two-sided Student’s *t*-test: ***p < 0.001. Source data are provided as a Source Data file.

In our assay, monocyte-derived macrophage cells THP-1 were first dual-labeled with Fluo-4 AM and CellROX DeepRed to simultaneously monitor the change of cytosolic calcium concentration and ROS level in real-time (Fig. 4a). To validate our assay, we treated our cells with lipopolysaccharides (LPS, 10 ng/mL), and we observed vigorous calcium transients and ROS production (Fig. 4c, d). To ensure that our results were not confounded by LPS contamination of the protein aggregate during the preparation of recombinant protein from bacterial cultures, the levels of LPS were quantified. (Supplementary Fig. 5b). The effective concentration of endotoxin in our tau samples when treating the cells was about 0.01–0.02 EU/mL, which was lower than our PBS buffer control.

To control for the variation in cell viability, signal traces were normalized to the basal intensity in each experiment, and ionomycin (1 μM) was perfused in the medium at the end of each trial as the positive control. Based on the results from our in vitro liposomal assay above (Fig. 3b), only s-tau aggregates taken at the 96 h timepoint were used and compared with WT tau fibrils in our subsequent investigation. Our data showed s-tau aggregates at 100 nM monomeric equivalent concentration caused strong characteristic peak-shaped calcium transients. As summarized in Fig. 4e, compared to WT aggregates which elicited calcium transients in 2.5 ± 2% of cells, self-assembled s-tau aggregates induced calcium transients in 85 ± 10% of the cells. The ROS level, on the other hand, gradually increased to 18 ± 10% upon the treatment of s-tau aggregates (Fig. 4b, Supplementary Fig. 6a). The application of WT tau aggregates, however, did not induce any significant changes in the ROS level (Fig. 4c, d). A similar null observation was also found in the s-tau monomer condition (Fig. 4c, d). In particular, the s-tau monomer control consisted of an equivalent amount of the tau protein and corresponding kinases and phosphorylation reaction buffer that contained a nanomolar range of ATP. The macrophage can respond to ATP in a concentration-dependent manner by activating purinergic receptors, providing a mechanism to perceive local cellular damage such as necrosis, which would release adenosine triphosphate into the extracellular domain. Therefore, the lack of response excluded the effect of purinergic receptors at the ATP concentration in our monomeric control, ensuring the macrophage activation triggered by s-tau observed was due to the presence of amorphous aggregates.

**Fig. 5:**
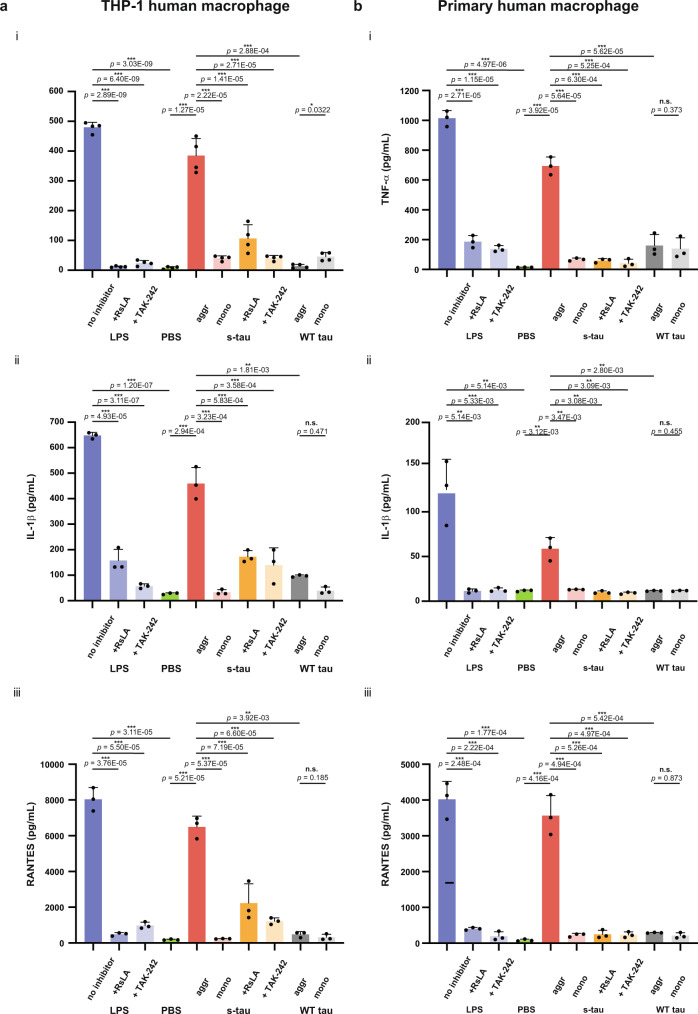
Hyperphosphorylated tau aggregates induced significantly higher levels of all three pro-inflammatory cytokines than heparin-induced WT aggregates. (i) TNF-a, (ii) IL-1β, and (iii) CCL5 ELISA assays were conducted on both **a** THP-1 human macrophage cells and **b** primary human macrophage cells to determine the pro-inflammatory responses mediated treatment by LPS, s-tau monomer or aggregates or heparin-induced WT aggregates or its monomeric control with or without the TLR4 antagonists RsLA or Tak-242. LPS and PBS served as the positive and negative controls. The *P*-values are based on unpaired two-sided Student’s *t*-test: **p < 0.01, ***p < 0.001, n.s. non-significant. Data are presented as mean values ± s.d. of three independent experiments except for **a**-(i) which had four independent experiments. Source data are provided as a Source Data file.

To further test calcium transients/ROS production as early biomarkers of macrophage activation and, more importantly, to investigate the pro-inflammatory nature of these hyperphosphorylated tau aggregates, we performed enzyme-linked immunosorbent assay (ELISA) to measure the levels of pro-inflammatory cytokines triggered by different tau species in THP-1. To determine the optimal dosing condition, THP-1 cells were incubated with various monomeric equivalent concentrations of different tau aggregates and corresponding monomer samples for 24 h, and the level of TNF-α was then evaluated in the cell supernatant. As a positive control, various concentrations of LPS ranging from 10 pg/mL to 10 ng/mL were applied to elicit robust pro-inflammatory responses, resulting in the release of TNF-α in a dose-dependent manner as expected (Supplementary Fig. 7). Our results showed s-tau aggregates at 500 nM monomer equivalent concentration could induce 385 ± 20 pg/mL of TNF-α secretion. In comparison, the same concentration of WT aggregates and s-tau monomer only elicited 105 ± 40 pg/mL and 40 ± 8 pg/mL, respectively (Supplementary Fig. 7, Fig. 5). Hence, 500 nM monomeric equivalent concentration was chosen for the subsequent ELISA experiments.

We then conducted TNF-α, IL-1β, and CCL5 ELISA assays to gain a more comprehensive understanding of the breadth and extent of pro-inflammatory responses mediated by tau aggregates on THP-1 cells. LPS and PBS served as the positive and negative controls, respectively, to verify the responsiveness of human macrophage cells under our treatment procedure. As demonstrated in Fig. 5a, s-tau aggregates induced significantly higher levels of all three pro-inflammatory cytokines than heparin-induced WT aggregates and its monomeric control. To verify whether s-tau aggregates had similar pathological effects on primary cells, we also applied different tau species onto primary macrophages derived from humans and our ELISA results again illustrated that hyperphosphorylated tau aggregates (s-tau) caused inflammatory responses more effectively than WT aggregates and its monomeric control (Fig. 5b).

To ensure such pro-inflammatory responses were directly caused by tau aggregates rather than as a secondary event induced by cell death or pyroptosis due to the presence of protein aggregates, we performed a cell viability assay and lactate dehydrogenase (LDH) assay on THP-1 cells when treated with different tau samples. Our results showed that tau aggregates and monomers of both species did not cause significant cellular death or compromise the membrane integrity compared to PBS buffer control under our 24 h treatment scheme (Supplementary Fig. 5c, d). A similar null observation has also been made by others^38^ in which study LPS (up to 2 μg/mL) did not yield significant cytotoxicity to THP-1 cells. Together, these results corroborated our conclusion that s-tau aggregates can elicit inflammatory responses in human macrophages.

### Hyperphosphorylated tau aggregates induce TLR4-dependent inflammatory responses

TLR4, a member of the Toll-like receptor family, has recently been demonstrated to play a crucial role in pro-inflammatory responses and ROS production triggered by α-synuclein and Aβ in immune cells^39^. Our previous work also suggested that protofibrillar Aβ aggregates can induce temporary calcium transients in astrocytes and promote the production of inflammatory species via TLR4 signaling pathway^40^. Therefore, to explore the underlying molecular mechanism of tau-induced pro-inflammatory responses, we utilized TLR4 inhibitors *Rhodobacter sphaeroides* lipid A (RsLA)^41,42^ and TAK-242^43–45^ to test its involvement in our macrophage assay. To first test the effectiveness of these TLR4 inhibitors, we demonstrated that pre-incubation of RsLA (5 μg/mL) or TAK-242 (1 μM) effectively mitigated the calcium transients, ROS elevation, and pro-inflammatory cytokine secretion in THP-1 cells induced by TLR4 agonist LPS (Fig. 4c, d, Supplementary Fig. 6b, c and Fig. 5a). These TLR4 inhibitors also effectively abated the production of TNF-α, IL-1β, and CCL5 induced by LPS in primary human macrophages, further validating the efficacy of these TLR4 inhibitors (Fig. 5b). Subsequently, we incubated the culture with either RsLA or TAK-242 30 min prior to the application of different tau species. Our result showed both TLR4 inhibitors were able to significantly attenuate the calcium transients and ROS production in THP-1 macrophages (Fig. 4e, f, Supplementary Fig. 6b, c) and suppress the secretion of all three pro-inflammatory cytokines of interest (TNF-α, IL-1β, and CCL5) in both THP-1 and primary human macrophage cells (Fig. 5). These results suggested that the observed proinflammation responses caused by tau aggregates were dependent upon the TLR4 receptor.

**Fig. 6:**
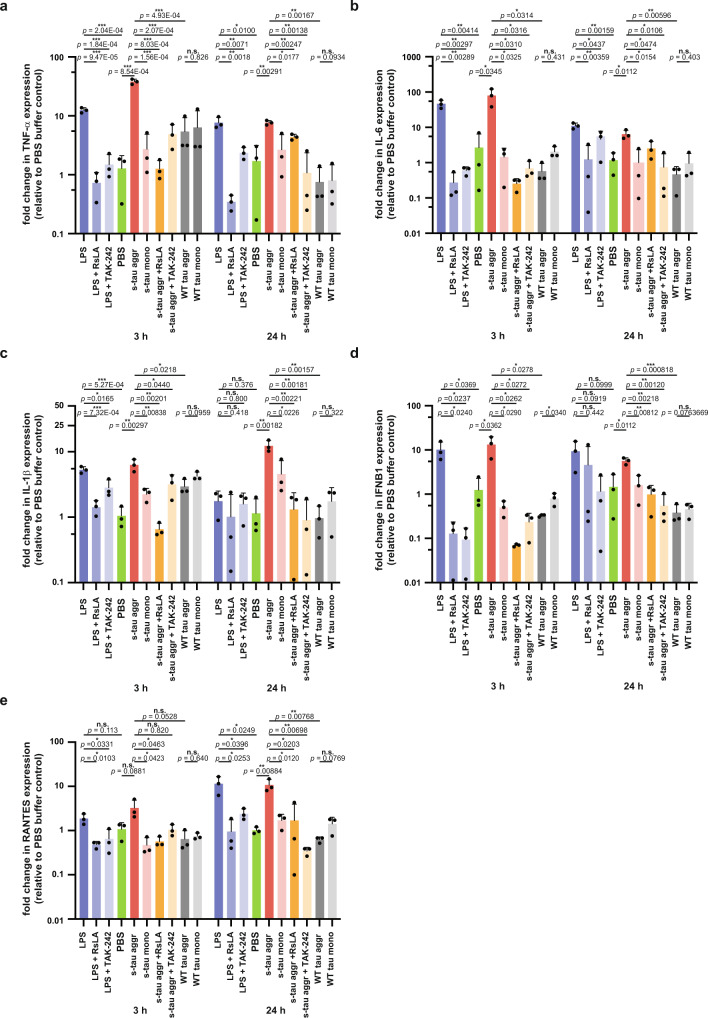
The expression of inflammatory-related genes *TNF-*α, *IL-6*, *IL-1*β, *IFN*β*1*, and *CCL5* were upregulated by hyperphosphorylated tau aggregates in THP-1 human macrophage and can be attenuated by TLR4 inhibitors. RT-qPCR measurements of the expression profile of inflammatory-related genes **a***TNF*-α, **b** *IL-6*, **c** *IL-1β*, **d** *IFNβ1*, **e** *CCL5* after 3 h and 24 h treatment by LPS, s-tau monomer or aggregates or heparin-induced WT aggregates or its monomeric control with or without the TLR4 antagonists RsLA or TAK-242. Positive and negative controls were LPS and PBS, respectively, and the data were normalized to the mean values of PBS gene expression for each gene of interest. The *P*-values are based on unpaired two-sided Student’s *t*-test: *p < 0.05, **p < 0.01, ***p < 0.001, n.s. non-significant. Data are presented as mean values ± s.d. of three independent experiments. Source data are provided as a Source Data file.

To further test the involvement of TLR4 we used reverse transcriptase quantitative PCR (RT-qPCR) to investigate the expression profile of inflammatory-related genes (*TNF-α*, *IL-6*, *IL-1β*, *IFNβ1*, and *CCL5*) that are downstream of the canonical TLR4 signaling pathways. As shown in Fig. 6, our results demonstrated that both NFκB target genes (*TNF-α*, *IL-6*, and *IL-1β*) and IRF-3 target genes (*IFNβ1* and *CCL5*) were upregulated after treatment with s-tau aggregates but not after treatment with s-tau monomer, WT tau aggregates and monomer, or when s-tau aggregate treatment was inhibited by TLR4 inhibitors (RsLA and TAK-242). Positive and negative controls were LPS and PBS, respectively, and the data were normalized to the mean values of PBS gene expression for each gene of interest. More specifically, at 3 h, *TNF-α*, *IL-6*, *IL-1β*, and *IFNβ1* were upregulated after s-tau aggregate treatment. At 24 h, all five target genes were upregulated following s-tau aggregate treatment, while *IL-1β* and *IFNb1* responses of THP-1 cells to LPS has no statistical difference compared to the PBS control. This suggests s-tau aggregates may have a prolonged effect on THP-1 cells, upregulating the expression of these inflammatory genes. The only occasion where there was no significant difference between s-tau aggregates and its monomeric control/inhibition conditions was the *CCL5* response at 3 h, but there was also no significant difference observed between LPS and PBS controls, thus suggesting that *CCL5* gene expression was not activated during the 3 h timeframe. Nonetheless, *CCL5* gene expression levels were elevated at the 24 h timepoint for both LPS and s-tau aggregate conditions.

The effectiveness of TLR4 inhibitors was demonstrated for each gene of interest: both antagonists RsLA and TAK-242 were able to block the action of LPS and reduce the gene expression of pro-inflammatory cytokines effectively and in most cases (37 out of 40 cases) to the basal levels. The only occasions where there was no significant difference between LPS and its inhibited conditions were *IL-1β* and *IFNβ1* response at 24 h, but those were when no significant difference between LPS and PBS control was observed. This suggests that *IL-1β* and *IFNβ1* response may have had passed their respective maximum response at 24 h, as LPS and its inhibited conditions had significant statistical differences for both *IL-1β* and *IFNβ1* response at 3 h. Thus, our transcriptomic results indicated that not only s-tau aggregates could trigger canonical TLR4 signaling pathways as both sets of NFκB target genes (*TNF-α*, *IL-6*, and *IL-1β*) and IRF-3 target genes (*IFNβ1*, *CCL5*) were upregulated but also TLR4 inhibitors could effectively mitigate the elevated pro-inflammatory gene expressions mediated by hyperphosphorylated tau aggregates in human macrophages.

## Discussion

### Hyperphosphorylated tau self-assembled to unstructured and amorphous aggregates

Since monomeric tau shows little intrinsic tendency for aggregation in vitro^3^, external inducers such as heparin and other polyanionic molecules have commonly been used to induce aggregation in vitro^46–48^. However, recent technological breakthroughs and developments of more sensitive biochemical assays suggest that heparin-induced tau and AD-derived tau have distinct conformations, properties, and bioactivities^15,16^. For instance, as illustrated in Fig. 1a, heparin can neutralize the positively charged amino acid sequences in microtubule-binding domains, thereby stabilizing R2 and R3 to form the dimeric core of heparin-induced aggregates^15^. In comparison, AD-derived tau aggregates are found to rather have R3 and R4 enclosing themselves to form the filamentous core^15,16^. Since these observations call the use of external inducers into question, more efforts have been focused on the impact of tau phosphorylation on its susceptibility to aggregation. At the molecular level, when monomeric tau is unmodified, the acidic N- and C-termini, as indicated by fluorescence resonance energy transfer and electron paramagnetic resonance experiments, fold over the vicinity of the microtubule-binding repeat domain, resulting in a paperclip conformation and thereby effectively preventing tau–tau intermolecular interaction^49^. Phosphorylation events, on the other hand, can cause an opening of such paperclip conformation^50^ and simultaneously stabilize α-helical structures, which is associated with the formation of tau aggregates^51^. Moreover, phosphorylation has been shown to affect not only the electrostatic properties^46^, but also local structures^52^ and the global fold of tau^53^. For instance, pseudophosphorylations (replacement of Ser and Thr residues by Glu residues) that reproduce AD-specific epitopes including AT8 (pSer^202^ and pThr^205^), AT100 (pThr^212^ and pSer^214^), and PHF-1 (pSer^396^ and pSer^404^) alone were able to induce tau monomer into the AD-specific conformation recognized by the conformational antibody MC-1^53^.

Here we have shown that sequential phosphorylation by PKA/GSK-3β or PKA/SAPK4 yielded hyperphosphorylated forms of tau with AD-specific epitopes that could self-assemble into small, amorphous aggregates. Similar to our observation, previous work showed that hyperphosphorylated tau (2N4R isoform) from Sf9 cells, which contains up to 20 phosphorylation sites on average and can oligomerize spontaneously without external inducer^19^. Our observations are also consistent with the current understanding^50^ that hyperphosphorylation can induce conformational changes at the monomeric level, as suggested by our SDS-PAGE results (Fig. 1b). According to a recent study ^54^, such differences in electrophoretic mobility are due not only to the different levels of phosphate group incorporation but more importantly to the different levels of SDS-resistant conformational changes induced by phosphorylation. Such structural reorganization of the molecular tau ultimately renders itself prone to aggregation, and more importantly, manifests as structural variations at the polymeric level, as evidenced by our morphological characterization of those different tau species (Fig. 2e–f, Supplementary Fig. 3b). Furthermore, a recent structural stimulation study shows the free-energy landscapes display two channels of tau aggregation^55^, with one leading to more ordered fibrils and the other to amorphous phases. Phosphorylation is shown to both thermodynamically and kinetically facilitate the amorphous aggregate channel, therefore supporting our observation that both hyperphosphorylated tau species polymerize into non-fibrillar structures. More importantly, a previous study identifies the formation of non-fibrillar tau aggregates—much similar to our observation of hyperphosphorylated tau aggregates—as an early sign of brain aging and AD, further bolstering the pathological relevance of our hyperphosphorylated tau synthesized in vitro^56^.

### Hyperphosphorylated tau aggregates trigger calcium influx of liposome assay suggesting toxicity via disruption of the lipid membrane

As mounting evidence suggests that mature filamentous deposits are not toxic, several studies begin to hint that non-fibrillar tau aggregates, an intermediate entity along the polymerization pathway, are more likely to be responsible for disease onset and progression^57^. In particular, hyperphosphorylated protofibrillar tau has been implicated in memory deficits, neuronal loss, nucleocytoplasmic trafficking disruption, tau propagation, and seeding both in vitro and in vivo^58,59^. Our membrane permeabilization liposome assay results are consistent with these previous studies demonstrating that smaller hyperphosphorylated tau aggregates are more efficient (with s-tau more efficient than g-tau) than heparin-induced mature filaments in disrupting lipid membranes. Several mechanistic frameworks have been proposed, hypothesizing that non-fibrillar tau structures might disrupt membrane integrity either by non-specific binding of amyloid oligomers to membrane surface^60^ or forming transmembrane oligomeric porelike structures^61^, or a combination of both. Previous studies have also indicated that such pore formation ability might be universally shared by other amyloidogenic proteins, including Aβ, α-synuclein, and prion-derived peptides besides tau, providing a more general mechanism that underlies the deleterious effects of all amyloid oligomers on lipid membrane and cell viability^62–64^.

### Hyperphosphorylated tau oligomers trigger elevated calcium signal and ROS production, elicit inflammation via TLR4 receptors in human macrophages

Although the spatial correlation between neuroinflammation and neurofibrillary tangle formation has been found in clinical data^65,66^, the causal relationship and mechanisms remain elusive. The present study showed that hyperphosphorylated tau aggregates could trigger TLR4-dependent pro-inflammatory reactions in human macrophages (both THP-1 cell line and primary human cells). We observed that most of the macrophages were able to remove the excess calcium triggered by hyperphosphorylated tau aggregates and recover to the basal level subsequently. This suggests that any membrane disruption or pore formation caused by the aggregates must have been transient, further supported by our results from LDH assay. Despite its transient nature, however, such calcium dysregulation has been shown to act as a secondary messenger and be able to potentiate TLR4-mediate response both in human macrophages and in vivo^67^. Previous studies have also demonstrated that TLR4 signaling in macrophages can enhance NADPH oxidase 2 (NOX2) activity which in turn promotes the production of ROS^68,69^ and stimulates the expression of pro-inflammatory cytokines such as IL-1β, IL-6, and CCL5^70^. Interestingly, calcium influx is also required for the activation of NOX2^71^. Hence, these earlier studies provide a potential mechanistic connection between calcium transient/ROS production of our immediate-early readouts and TLR4 activation as quantified by the cytokine expression and secretion. Moreover, tau aggregate-induced lipid membrane destabilizations as measured by the membrane permeabilization assay could also contribute to the calcium dysregulation observed in neurons and glial cells of AD brain^72^ and hence be an additional mechanism of toxicity in the cellular environment. Our results do not necessarily preclude the possibility that various mechanisms work synergistically to exert potential cellular damage. In future work it would be of interest to determine if human neurons and astrocytes also respond to tau aggregates and if the response is mediated by TLR4. Such studies will help determine if our observations are confined to only myeloid cells or would it be applicable to other cell types in the brain. In summary, our current work provides a potential mechanistic insight linking hyperphosphorylation-driven tau aggregation with neuroinflammation and neurodegeneration. These hyperphosphorylated tau aggregates are shown to be cytotoxic by diverse mechanisms, including destabilization of lipid membranes and TLR4-dependent macrophage inflammation. These mechanisms could contribute to the disruption of calcium homeostasis, induction of oxidative stress, and release of pro-inflammatory cytokines observed in tauopathies. It therefore becomes important to now establish if cytotoxic hyperphosphorylated tau aggregates, similar to those characterized in this work, are detected in tauopathies. This work may prove important for developing therapeutics for AD and other tauopathies, by either preventing the formation of hyperphosphorylated tau aggregates or reducing their neurotoxic effects.

## Methods

### Protein expression and purification

Human 0N4R tau was expressed as previously described^26^. In brief, pRK172 plasmids (gift from Michel Goedert) expressing full-length tau (isoform 0N4R) wild-type were transformed in BL21(DE3) cells. Transformed *E. coli* BL21 cells were grown in Luria Broth media containing 100 μg/mL ampicillin at 37 °C under shaking conditions until OD_600_ of 0.6 was reached, and tau expression was induced by the addition of 1 mM IPTG for 4 h. Cells were then harvested by centrifugation at 4000 × *g* (JA-20 rotor, Beckman Coulter) for 30 min at 4 °C, resuspended in lysis buffer (50 mM MES [pH 6.0] with 2,5 mM TCEP, 1 mM AEBSF), and lysed using a probe sonicator (1 × 1.5 min, 5 s on, 10 s off, 40% amplitude). The cell debris was subsequently removed by centrifugation for 30 min at 18,000 × *g* at 4 °C. RNase and DNase were added before the supernatant was filtered and loaded onto a Resource S ion exchange column (GE Healthcare). Protein was eluted over a linear NaCl gradient from 0 to 500 mM, and fractions containing tau protein (determined by SDS-PAGE) were pooled and precipitated with 20% ammonium sulfate at 4 °C for 1 h. The protein was pelleted at 15,000 × *g* for 20 min at 4 °C. After the pellet was resuspended in SSPE buffer with 2.5 mM TECP and 0.1 mM PMSF, the protein was further purified using size-exclusion chromatography with a Superdex 200 Increase 10/300 size-exclusion column (GE Healthcare Life Sciences). The fractions were collected and analyzed by SDS-PAGE, and those having the purest bands corresponding to tau were pooled. The protein concentration was then determined by measuring its absorbance at 280 nm on a Nanodrop 2000 using an extinction coefficient of 7450 M^−1^ cm^−1^ and by BCA assay (Thermo Fisher) following the manufacture protocol. Aliquots were flash-frozen in liquid nitrogen and stored at −80 °C.

### In vitro phosphorylation of recombinant tau

Full-length recombinant tau (0N4R) was sequentially phosphorylated as described previously^10^. Monomeric tau was incubated at a final concentration of 50 μM in 25 mM Tris-HCl buffer [pH 7.4] (Sigma–Aldrich) with 0.1 mM ethylene glycol tetraacetic acid (EGTA, Sigma–Aldrich), 2 mM AEBSF protease inhibitor (Sigma–Aldrich), 10 mM magnesium acetate, 2 mM ATP (Sigma–Aldrich). 0.018 U of the catalytic subunits of cAMP-dependent protein kinase A (PKA, BioVision) per nmol of tau was added and the reaction was incubated at 30 °C. ﻿After 24 h, the second kinase (GSK-3β or SAPK4 from Abcam, 0.018 U per nmol of tau) was added and the reaction proceeded for an additional 24 h. One unit of the kinase is defined as the amount of enzyme that will transfer one nmol phosphate from ATP to the corresponding substrate per min at pH 7.4 at 30 °C, tested and reported by individual manufacturers. Phosphorylation was confirmed in sodium dodecyl sulfate-polyacrylamide gel electrophoresis (SDS-PAGE). Residual LPS is also known as endotoxin derived from the outer cell membrane of gram-negative bacteria can elicit TLR4 signaling. To ensure that our results were not confounded by the residual LPS in our tau proteins purified from bacterial cultures, LPS contamination was effectively eliminated after phosphorylation reactions by using high-capacity endotoxin removal spin columns (Thermo Fisher). This column has been shown to effectively remove contaminating endotoxins (i.e., residual lipopolysaccharides) in protein samples purified from bacterial cultures without introducing other bioactive species. Our endotoxin quantification results subsequently indicated that at 2 μM WT tau contained 0.47 ± 0.05 EU/mL, g-tau, 0.37 ± 0.13 EU/mL, and s-tau 0.43 ± 0.25 EU/mL with PBS buffer control 0.13 ± 0.03 EU/mL. After endotoxin removal, the protein concentration was then determined by Nanodrop and by BCA assay as mentioned above. Aliquots at 40 μM were flash-frozen in liquid nitrogen and stored at −80 °C.

### Liquid chromatography-tandem mass spectrometry (LC-MS/MS)

Detailed phosphorylation mapping was obtained from LC-MS/MS analysis conducted by Cambridge Center for Proteomics, and the measurements were repeated across three independent experiments for both g-tau and s- tau proteins to ensure reproducibility. Phosphorylation sites observed across three repeats were highlighted in Supplementary Table 1 for each species. Variations due to the behavior of the tryptic peptides in the instrument were minimal.

Protein solutions were reduced (DTT), alkylated (iodoacetamide), and enzymatically digested in 50 mM ammonium bicarbonate [pH 8] with trypsin overnight at 37 °C. After digestion, the supernatant was pipetted into a sample vial and loaded onto an autosampler for automated LC-MS/MS analysis.

All LC-MS/MS experiments were performed using a Dionex Ultimate 3000 RSLC nanoUPLC (Thermo Fisher Scientific Inc, Waltham, MA, USA) system and a QExactive Orbitrap mass spectrometer (Thermo Fisher Scientific Inc, Waltham, MA, USA). Separation of peptides was performed by reverse-phase chromatography at a flow rate of 300 nL/min and a Thermo Scientific reverse-phase nano Easy-spray column (Thermo Scientific PepMap C18, 2 μm particle size, 100 A pore size, 75 μm i.d. × 50 cm length). Peptides were loaded onto a pre-column (Thermo Scientific PepMap 100 C18, 5 μm particle size, 100 A pore size, 300 μm i.d. × 5 mm length) from the Ultimate 3000 autosampler with 0.1% formic acid for 3 min at a flow rate of 10 μL/min. After this period, the column valve was switched to allow the elution of peptides from the pre-column onto the analytical column. Solvent A was water + 0.1% formic acid and solvent B was 80% acetonitrile, 20% water + 0.1% formic acid. The linear gradient employed was 2–40% B in 30 min.

The LC eluant was sprayed into the mass spectrometer by means of an Easy-Spray source (Thermo Fisher Scientific Inc.). All m/z values of eluting ions were measured in an Orbitrap mass analyzer, set at a resolution of 35,000, and was scanned between *m/z* 380 and 1500. Data-dependent scans (Top 20) were employed to automatically isolate and generate fragment ions by higher-energy collisional dissociation (HCD, NCE:25%) in the HCD collision cell and measurement of the resulting fragment ions was performed in the orbitrap analyzer, set at a resolution of 17,500. Singly charged ions and ions with unassigned charge states were excluded from being selected for MS/MS and a dynamic exclusion window of 20 s was employed.

Post-run, all MS/MS data were converted to mgf files, and the files were then submitted to the Mascot search algorithm (Matrix Science, London UK) and searched against the UniProt Human database (93,609 sequences; 37,041,084 residues) and a common contaminant sequence containing non-specific proteins such as keratins and trypsin (125 sequences; 41,129 residues). Variable modifications of oxidation (M), deamidation (NQ), and phosphorylation (S,T,Y) were applied as well as fixed modification of carbamidomethyl (C). The peptide and fragment mass tolerances were set to 20 ppm and 0.1 Da. A significance threshold value of *p* < 0.05 and a peptide cutoff score of 20 were also applied.

### High-resolution native mass spectrometry

The protein samples were stored in phosphorylation reaction buffer at −80 °C before being buffer exchanged into 200 mM ammonium acetate [pH 6.8] by multiple rounds of concentration and dilution using the Pierce^TM^ protein concentrators (Thermo Fisher). Finally, the samples were diluted to a monomer equivalent concentration of 1 mM before the measurements. The data was collected using in-house gold-plated capillaries on a Q Exactive^TM^ mass spectrometer in positive ion mode with a source temperature of 70 °C and a capillary voltage of 1.2 kV. In-source trapping was set to −120 V to help with the dissociation of small ion adducts. Ion transfer optics and voltage gradient throughout the instruments were optimized for ideal transmission. Spectra were acquired with 10 microscans to increase the signal-to-noise ratio with transient times of 64 ms, corresponding to the resolution of 17,500 at *m/z* = 200, and AGC target of 1.0 × 10^6^. The noise threshold parameter was set to 3 and the scan ranges used were 346–14,147 and 1000–10,000 *m/z* for g- and s-tau, respectively.

The measurements were repeated across three independent experiments for both g-tau and s- tau proteins to ensure reproducibility. Mass deconvolution was achieved using the UniDec software as previously described^73^. Before deconvolution, the data were pre-processed by setting the curved subtraction to ten, which resulted in significantly more baseline-resolved peaks. Further, the fitted peak FWHM was optimized for each spectrum (generally within 0.5 and 1 Th). All other UniDec parameters were left at their default values. The deconvolution was performed in the mass range of 40–44 kDa. The estimation of the degree of phosphorylation was complicated by small ion adducts present in the deconvoluted mass spectra, which extend the range of masses associated with any particular phosphorylation state, as well as possibly cause some mass overlap. To find the optimal distribution of phosphate groups that explained the deconvoluted mass spectrum, as well as the uncertainty of this assignment, a Markov Chain Monte Carlo assignment method was employed. This was done using the emcee python package as previously described^74^ ([1202.3665] emcee: The MCMC Hammer (arxiv.org)). The masses of one phosphate group and one small ion adduct were set to 80 and 23 Da, respectively. The mass of the small ion adduct was chosen so that it is within the integration window of 10 Da. Two most probable adducts were the ammonium and sodium cations. The priors used for fitting were the number of the phosphate groups and small ion adducts which were kept below 50, independently. The limits were chosen for time efficiency and to keep make sure the combined mass of the phosphate groups was not likely to go above the 44 kDa limit of the deconvolved mass spectrum. Six chains were run for 3000 steps each: the first 800 steps were discarded as a burn-in, and the rest was thinned by a factor of 100 to reduce any autocorrelation. The thinned chains were then used to estimate the extent of phosphorylation by summing the area under the curve (to account for the inherent broadness of the deconvolved mass peaks, as well as the fact that there are two possible small adduct ions) in corresponding mass regions using a mass window of 10 Da.

### Standard tau aggregation procedure

Unphosphorylated and phosphorylated tau protein was used. Monomers were incubated at 2 μM in PBS buffer (137 mM NaCl, 3 mM KCl, 8 mM Na_2_HPO_4_, 1.5 mM KH_2_PO_4_, pH 7.3) with 0.01% NaN_3_ at 37 °C. Heparin (Fisher Scientific) was added at 10 μg/ mL (about 2 μM) to induce aggregation. Self-assembly reactions were rather carried out in the absence of heparin.

### SDS-PAGE

Protein samples along with molecular weight marker (SeeBlue™ Plus 2 Pre-Stained Standard) were loaded into the wells of 4–12% gradient NuPAGE Bis-Tris precast gels or 10% self-cast SDS-PAGE gels and run in MOPS-based SDS running buffer for 55 min at 200 V. The proteins were visualized by Coomassie stain.

### Single-molecule TIRF microscopy

#### Instrumentation

The samples were imaged using a home-built total internal reflection fluorescence (TIRF) microscope. The total internal reflection mode limits the fluorescence field to 200 nm from the sample slide. For pFTAA imaging, a 488 nm diode laser (TOPTICA iBeam smart, 175 mW) was aligned and directed to the optical axis at the edge of a TIRF objective (Apo TIRF 100 × /NA1.49 oil objective, Nikon) mounted on an inverted microscope (Eclipse T*i*-S, Nikon). The emitted fluorescence was collected by the same objective, separated from the returning TIRF beam by a dichroic mirror (Di01-R405/488/532/635-25 × 36, Semrock), and passed through an emission filter (FF552-Di02-25 × 36, Semrock). For ThX super-res imaging, the same 488 nm laser was used, but a different set of emission filters (LP02-568RS-25, FF01-587/35-25, Semrock) was utilized to acquire the optimal super-resolution images. The control of the hardware was enabled by custom-written scripts for MicroManager (NIH). The signal was recorded on an Evolve Delta 512 EMCCD camera (Photometrics) Each pixel was 107.2 nm in length such that the dimensions of a single image taken were 54.8 by 54.8 μm^2^.

The switch from TIRF to widefield mode was accomplished by altering the beam offset perpendicular to the optical axis. To simultaneously track calcium transients and ROS level changes, a 488 nm diode laser (TOPTICA iBeam smart, 175 mW) and a 638 nm laser (Cobolt 06-MLD, 180 mW) were aligned and directed to the optical axis of a ×20 objective (Nikon) mounted on an inverted microscope (Eclipse T*i*-S, Nikon). The emitted fluorescence was collected by the same objective, separated from the returning beam by a dichroic mirror (Di01-R405/488/532/635-25 × 36, Semrock), and passed through an emission filter (FF580-FDi01-25 × 36, Semrock for 488 nm excitation and BLP01-635R-25, Semrock for 638 nm excitation). Each channel was recorded alternatingly at a 1 s temporal resolution, and each frame had an exposure time of 100 ms.

#### Sample preparation for TIRF imaging

Prior to imaging, Borosilicate glass coverslips (Ø50 mm, VWR International) were cleaned using an argon plasma cleaner (PDC-002, Harrick Plasma) for 1 h to remove any autofluorescent residue. Multiwell slide chambers (CultureWell chambered cover glass 50 well, Grace Bio-Laboratories) were affixed onto the cleaned coverslips. To stain the aggregates for diffraction-limited imaging, samples were diluted in PBS buffer containing 30 nM pFTAA (gift from Michel Goedert) to a final monomer equivalent concentration of 67 nM tau. Then 15 μL of each sample was applied to each well on the coverslip for 15 min before imaging. Images were recorded for 100 frames, each with an exposure time of 50 ms. The image stacks were averaged using ImageJ (NIH) software for further analysis.

To super-resolve the structures of tau aggregates, especially the early aggregate species whose lengths are under 100–200 nm, ThX, a synthetic variant of well-documented Thioflavin T (ThT), was used. After the coverslips being plasma cleaned, the surface was washed with PBS buffer and coated with poly-L-lysine (PLL, 1 mg/mL) for 15 min to reduce non-specific binding events. After gently removing the excess PLL coating, gold nanoparticles with 200 nm diameter size were applied onto the surface for 5 min, serving as fiducial markers to correct thermal drift during imaging. The surface was then incubated with protein aggregates for 20 min. Subsequently, samples were imaged in the presence of 1 μM ThX dye dissolved in PBS buffer. Note that one or two washes with PBS between each surface treatment procedure, i.e., PLL coating, gold nanoparticles application, and protein incubation, are recommended to obtain the optimal imaging condition. Images were recorded for 10,000 frames, each with an exposure time of 20 ms.

#### Diffraction-limited image analysis

For each sample, 16 fields of view were typically recorded. Individual image data were averaged overall the framed by ImageJ (NIH) software and then analyzed with a custom-written MATLAB script as previously described^75^ (R2018b, MathWorks) to count the total number of fibrils and aggregates, the size, and fluorescence intensity. Since both the concentrations of protein and pFTAA were controlled in each experiment, the number and the intensity of fibrils and aggregates can be directly compared across different experiments. For particle identification, images were bandpass filtered to remove the modulated background and camera noise. To identify particle boundaries, the foreground was then blurred using a 2D-Gaussian filter with a threshold applied based on the pixel intensity with a criterion of 2% intensity above the background (median value of the whole image). The length of aggregates was measured by thinning boundaries of individual particles and then calculated with an image pixel size of 107.2 nm for our TIRF setup. To eliminate the background effect in our intensity calculation, signal-to-background ratio (SBR) was introduced to correct the intensity of pixels, where the SBR is defined as:

1 $${{{{{{\mathrm{SBR}}}}}}}=\frac{{{{{{{\mathrm{Intensity}}}}}}}\,{{{{{{\mathrm{above}}}}}}}\,{{{{{{\mathrm{the}}}}}}}\,{{{{{{\mathrm{background}}}}}}}}{{{{{{{\mathrm{Background}}}}}}}}$$

For a given particle, its corrected intensity is the sum of each pixel’s SBR values within its boundary.

#### Super-resolution image analysis

The raw image stacks were passed through a custom-written Python script as previously described^76^. We defined a localization as 2D-Gaussian fit of a fluorescence signal in one image frame. The localizations were determined using the ‘Peak Fit’ plugin within Fiji (ImageJ)/GDSC Single Molecule Light Microscopy package (http://www.sussex.ac.uk/gdsc/intranet/microscopy/UserSupport/AnalysisProtocol/imagej/gdsc_plugins/). The adjustable parameters ‘signal strength’ and ‘precision’ were set to 3 and 30 nm, respectively. These values referred to the properties of the 2D-Gaussian fit and were set by visual inspection of the rendered localization images. The fiducial markers of the images were pinpointed as localizations that lasted more than 500 frames at the same location. Fiducial signals were first removed from the localization file from the subsequent analysis.

Then image stacks went through temporal grouping, which sorted localizations into bursts. Temporal grouping was achieved by (1) using DBSCAN clustering function (scikit-learn) on the spatial domain (XY coordinates) of the localizations, with a detection radius (epsilon) of 15 nm and minimum point threshold of 3. (2) using DBSCAN again on the temporal domain (frame number) with an epsilon of 21 ms and a minimal frame number of 2 to recognize individual bursts and remove single frame localizations. The rationale of the settings here is that many non-specific binding events are characterized by short transient signals that last less than the exposure time of the camera. Finally, the burst information was processed through a final DBSCAN cluster analysis on the spatial domain using an epsilon of 200 nm and a minimal burst number of 20 to group the bursts into clusters, which were interpreted as detections of individual super-resolved protein aggregates.

For each cluster analyzed, the number of bursts ranged from 34 to 3356. Subsequently, we structurally characterized the sizes of clusters by (1) scaling the spatial domain by eight times and rounding the coordinates to integers; (2) closing the morphological space between bursts using the scikit-image ‘closing’ function; (3) skeletonizing the closed shape to a width of a single pixel using the scikit-image ‘skeletonize_3d’ function; (4) calculating the lengths by traversing the skeleton and recording the 8-connectivity distances. Finally, the clusters were loaded into the ‘Results Manager’ plugin in Fiji/GDSC SMLM and rendered into super-resolution images with each cluster labeled and characterized.

#### Fourier ring correlation analysis

The resolution was determined by plotting a Fourier ring correlation curve with the FIRE plugin^77^ for ImageJ/Fiji and the spatial frequency was determined at which the curve drops below 1/7.

### In vitro membrane permeabilization assay

Liposomal penetration assay was conducted as described previously^30^. To prepare the lipid vesicles, 16:0–18:1 PC (10 mg/mL) and 18:1–12:0 Biotin PC (1 mg/mL) (Avanti Lipids) were mixed with 100:1 ratio. The lipid mixture was then hydrated in HEPES buffer (50 mM, pH 6.5) with 100 µM Cal-520 (Stratech). Five freeze-and-thaw cycles were performed using a water bath and dry ice to acquire the unilamellarity. The lipid solution was extruded at least 10 times through a membrane with a size cutoff of 200 nm, and the size of the vesicles was measured using a Zetasizer (Zetasizer Nano ZSP). Free dyes were removed using size-exclusion chromatography.

To prepare the surface, glass coverslips (VWR International, product number 63 1-0122) were cleaned by sonicating in 2% (v/v) Hellmanex III (Hellma GmbH & Co. KG) in Milli-Q water for 10 min followed by sonicating twice in Milli-Q water and methanol for 10 min, respectively. Then the coverslips were dried under a stream of nitrogen gas, and plasma-etched using an argon plasma cleaner (PDC-002, Harrick Plasma) for 1 h to remove any fluorescent impurities. Each cover slide was fixed by frame-seal incubation chambers (Biorad, Hercules) and the surface was coated with 100:1 PLL-g-PEG and PLL-g-PEG biotin (SuSoS AG) (1 g/L) in 50 mM HEPES buffer. Then the coverslips were washed three times and 0.1 mg/mL neutravidin (Thermo Scientific) solution was added to the coverslips and incubated for 15 min and washed three times with reaction buffer. Then, a 50 µL aliquot of the solution of purified biotinylated vesicles was added to the surface and incubated for 30 min before washing carefully at least five times with reaction buffer. Single vesicles tethered to borosilicate glass cover slides via biotin neutravidin linkage were incubated with 50 µL Ca^2+^ containing buffer solution Leibovitz’s L-15 (phenol red-free) and a background image was recorded (F_background_). Fifty microliters of the sample was added, images were acquired (F_sample_), and care was taken to avoid moving the glass coverslips during the addition of each sample. Next, 10 µL of a solution containing 1 mg/mL of ionomycin, an ionophore for Ca^2+^ ion, and subsequently images of Ca^2+^ saturated single vesicles in the same fields of view were acquired (F_ionomycin_). For each field of view, 50 images were taken with an exposure time of 50 ms. The fields of view were chosen using an automated program via ImageJ Micromanager. The relative influx of Ca^2+^ into an individual vesicle due to aggregates of tau was then determined as

2 $${{{{{\rm{Percent}}}}}}\,{{{{{\rm{of}}}}}}\,{{{{{{\rm{Ca}}}}}}}^{2+}{{{{{\rm{influx}}}}}}=\frac{{F}_{{{{{{\mathrm{sample}}}}}}}-{F}_{{{{{{\mathrm{background}}}}}}}}{{F}_{{{{{{\mathrm{ionomycin}}}}}}}-{F}_{{{{{{\mathrm{background}}}}}}}}$$

The average calcium influx was calculated by averaging the Ca^2+^ influx into individual vesicles in each field of view.

### TEM

To prepare samples for TEM experiments, protein solutions at an appropriate concentration (typically ~200 nM) were applied onto a carbon-coated 400-mesh copper grid (Agar Scientific) for 1 min, and then stained with 2% (v/v) uranyl acetate for another minute. The excess solution was removed from the grid by washing twice with Milli-Q water. After samples dried thoroughly, TEM images were acquired using Thermo Scientific (FEI) Talos F200X G2 microscope (Department of Chemistry, Cambridge) operated at 200 kV.

### Live imaging of human macrophage THP-1 cells

Human monocytic THP-1 cells (ATCC-TIB-202 from LGC Ltd, Middlesex, UK) were cultured in Roswell Park Memorial Institute medium (RPMI-1640, Invitrogen) culture medium supplemented with 10% of heat-inactivated fetal bovine serum (Invitrogen) in 5% CO_2_ humidified atmosphere at 37 °C. THP-1 monocytes were first seeded at 200,000 cells/mL on sterilized glass coverslips. Then THP-1 cells were differentiated into macrophages in the presence of 20 ng/mL phorbol 12-myristate 13-acetate (PMA, Sigma–Aldrich) for 3 days continuously. This was followed by a recovery period of 24 h in serum-supplemented RPMI-1640 medium without PMA. Cell differentiation was verified by evaluating cell adhesion and spreading under an optical microscope. For Fluo-4 AM and ROX epifluorescence imaging, differentiated THP-1 cells are washed three times with OPTI-MEM and then incubated with 7.5 μM Fluo-4 AM and 6.25 μM CellROX Deep Red Reagent in OPTI-MEM for 30 min. To remove the free dye thoroughly, the cells are washed with OPTI-MEM three times before imaging. CellROX DeepRed reagent, weakly fluorescent while in a reduced state and exhibits photostable fluorescence upon oxidation by reactive oxygen species (ROS), is a proprietary reagent from Life Technologies. CellROX Deep Red has been ﻿reported to robustly detect a wide range of pro-oxidants, including superoxide anion, hydroxyl radical, peroxynitrite, and to a lesser extent, nitric oxide^78,79^.

### Primary human macrophage cell culture

Blood samples were donated by healthy volunteers who had undertaken informed consent in accordance with local Research Ethics Committee approval. Peripheral blood mononuclear cells were isolated from citrated peripheral blood samples by density gradient separation using Lympholyte (Cedarlane Labs), and subsequent CD14+ positive selection using the MACS Miltenyi Biotec Human CD14 microbead protocol (Miltenyi Biotec). CD14+ cells (3 × 10^5^ cells per well) were differentiated into macrophages using recombinant human granulocyte-macrophage colony-stimulating factor (200 ng/mL GM-CSF) and recombinant human interferon gamma (50 ng/ml IFNg) (Peprotech) in standard tissue culture DMEM media containing 10% fetal calf serum.

The human primary monocytes were extracted from the blood from donors who gave their samples voluntarily without any compensation. They gave informed consent via a Good Clinical Practice registered member of staff from Royal Papworth Hospital NHS Foundation Trust Cambridge under local ethics committee approval (REC No. 12/WA/0148). No human-derived cells were stored after the experiments had been completed.

### Macrophage calcium and ROS signal processing

Data were analyzed in MATLAB (MathWorks) using custom software. Cells were located by tracking local maxima and the intensity over time of a cell-sized disk surrounding the mean position of each cell was analyzed. The code is read in a single TIFF file representing a 3D time series. After background subtraction, flat-field correction, and Gaussian smoothing, local maxima in each frame were identified. Maxima from each frame were combined into tracks using a nearest-neighbor approach. If the standard deviation of the position was greater than half the cell radius, the track would be discarded. Otherwise, the mean position was assumed to be the location of a cell. The mean intensity of a cell-sized disk around each of these fixed positions was calculated, forming intensity traces for each cell. The noise was reduced by Gaussian smoothing. Sharp increases in calcium intensity were then identified from the first derivative and a user-defined threshold determined which of these were classified as calcium spikes. If a minimum spike intensity and/or minimum spike duration have been specified at the start, spikes not meeting these criteria were discarded. The percentage of ROS signals changed was calculated as:

3 $${{{{{\rm{Percent}}}}}}\; {{{{{\rm{of}}}}}}\; {{{{{\rm{ROS}}}}}}\; {{{{{\rm{signals}}}}}}\; {{{{{\rm{changed}}}}}}=\frac{{F}_{{{{{{{\mathrm{final}}}}}}}}-{F}_{{{{{{{\mathrm{initial}}}}}}}}}{{F}_{{{{{{{\mathrm{initial}}}}}}}}}$$

The initial intensity was defined by averaging the DeepRed fluorescence intensities across 50 frames prior to the addition of tau aggregates, and the final intensity was determined by averaging the DeepRed fluorescence intensities across 50 frames prior to the addition of ionomycin. Further details and the source code are available at https://github.com/janehumphrey/calciumStationaryCells.

### Cell viability measurements

Cell viability was assessed using Live-or-Dye^TM^ fixable viability staining kit (Biotium, Live-or-Dye^TM^ 750/777), following manufacture protocol. Briefly, human monocytic THP-1 cells (ATCC) were cultured and differentiated into macrophages as described above. After being treated with different tau species of 500 nM monomeric equivalent concentration for 24 h, cells were harvested using TrypLE^TM^ Express Enzyme (Thermo Fisher). Cells were subsequently pelleted by centrifugation at 350 × *g* for 5 min and the supernatants were discarded. Cells were washed once in PBS and then resuspended in PBS at 1 × 10^6^ cells/mL, followed by incubation at room temperature with the viability stain (diluted 1:1000 in PBS) for 30 min. Afterward, cells were washed and resuspended in PBS and then analyzed by flow cytometry in the 633 nm channel.

### LDH assay

Levels of lactate dehydrogenase (LDH) were measured by CyQUANT^TM^ LDH cytotoxicity assay kit (Invitrogen) as an indicator of cytotoxicity, following manufacturing protocol. Briefly, human monocytic THP-1 cells (ATCC) were cultured and differentiated into macrophages as described above. After being treated with different tau species of 500 nM monomeric equivalent concentration for 24 h, 50 μL of conditioned media of each sample along with maximum LDH activity controls (generated using 10× lysis buffer) and spontaneous LDH activity controls (no treatment group) were incubated with 50 μL LDH reaction mixture in a 96-well flat-bottom plate at room temperature for 30 min. Afterward, 50 μL of stop solution was added to each sample well to quench the reaction. Absorbance was measured at 490 nm and 680 nm: before calculating the percentage of toxicity, the 680 nm absorbance value (background) must be subtracted from 490 nm absorbance, and then the percentage of cytotoxicity can be determined using the following formula:

4 $${{{{{\rm{Percent}}}}}}\; {{{{{\rm{of}}}}}}\; {{{{{\rm{cytotoxicity}}}}}}=\frac{{{{{{{{\mathrm{LDH}}}}}}}}_{{{{{{{\mathrm{sample}}}}}}}}-{{{{{{{\mathrm{LDH}}}}}}}}_{{{{{{{\mathrm{spontaneous}}}}}}}}}{{{{{{{{\mathrm{LDH}}}}}}}}_{{{{{{{\mathrm{maximum}}}}}}}}-{{{{{{{\mathrm{LDH}}}}}}}}_{{{{{{{\mathrm{spontaneous}}}}}}}}}$$

### Enzyme-linked immunosorbent assay (ELISA)

The levels of TNF-α, IL-1β, and CCL5 secreted into the cell supernatant were measured by Abcam ELISA kits respectively (ab181421; ab214025; ab174446) following the manufacture protocol. Ultrapure LPS *E. coli* O111:B4 (Cayman Chemical, CAY-28872-10mg) was used as a positive control to elicit robust TLR4 activation, while small molecule inhibitors TAK-242 (Tocris Bioscience) and Ultrapure RsLA (InvivoGen) were employed to block TLR4 activation and therefore to validate the involvement of TLR4 receptor. Total protein concentrations were then determined by Pierce BCA assay following the manufacture protocol to ensure the total protein concentrations were uniform and controlled across different treatment groups.

### Reverse transcriptase quantitative PCR (RT-qPCR)

Total RNA was isolated from differentiated THP-1 macrophages, using the Monarch^®^ total RNA miniprep kit (New England BioLabs). Two hundred nanograms of total RNA was used in the cDNA synthesis reaction with the High-Capacity RNA-to-CDNA kit (Thermo Fisher Scientific), and the resulting cDNA was subsequently used to analyze gene expression on a QuantStudio 5 qPCR System (Thermo Fisher Scientific) by TaqMan gene expression assays for TNFα (Hs00174128_m1), IL-6 (Hs00174131_m1), IL-1β (Hs01555410_m1), IFNb1 (Hs01077958_s1), and CCL5 (Hs99999048_m1). Amplification parameters were 50 °C for 2 min and then 95 °C for 10 min, followed by 40 cycles of 95 °C for 15 s alternating with 60 °C for 60 s. Relative Gene expression was determined based on the ΔC_t_ values between the gene of interest and housekeeping genes GAPDH (Hs02786624_g1) and 18 S rRNA (Hs03003631_g1) compared with the mean ΔC_t_ values for the PBS buffer control group.

### Reporting summary

Further information on research design is available in the Nature Research Reporting Summary linked to this article.

## Supplementary information

Supplementary Information

Reporting Summary

## Data Availability

The mass spectrometry proteomics data in this study have been deposited to the ProteomeXchange Consortium via the PRIDE partner repository with the accession code PXD033127 (Human tau 0N4R isoform hyperphosphorylation LC-MS/MS). The remaining data are available within the Article, Supplementary Information, or Source Data file. Source data are provided with this paper.

## Source data

Source Data

## Code availability

The super-resolution analysis code [10.5281/zenodo.4651484] is available at https://github.com/Eric-Kobayashi/SR_toolkit. The source code for the calcium/ros analysis [10.5281/zenodo.6390919] is available at https://github.com/janehumphrey/calciumStationaryCells.
