# Supplementary material for: Hyperphosphorylated tau self-assembles into amorphous aggregates eliciting TLR4-dependent responses
Source: Nat Commun. 2022 May 16;13:2692. doi: 10.1038/s41467-022-30461-x (PMC9110413; doi:10.1038/s41467-022-30461-x)
Supplement: Supplementary file 1 — Supplementary Information [file 41467_2022_30461_MOESM1_ESM.pdf]

Supplementary Information

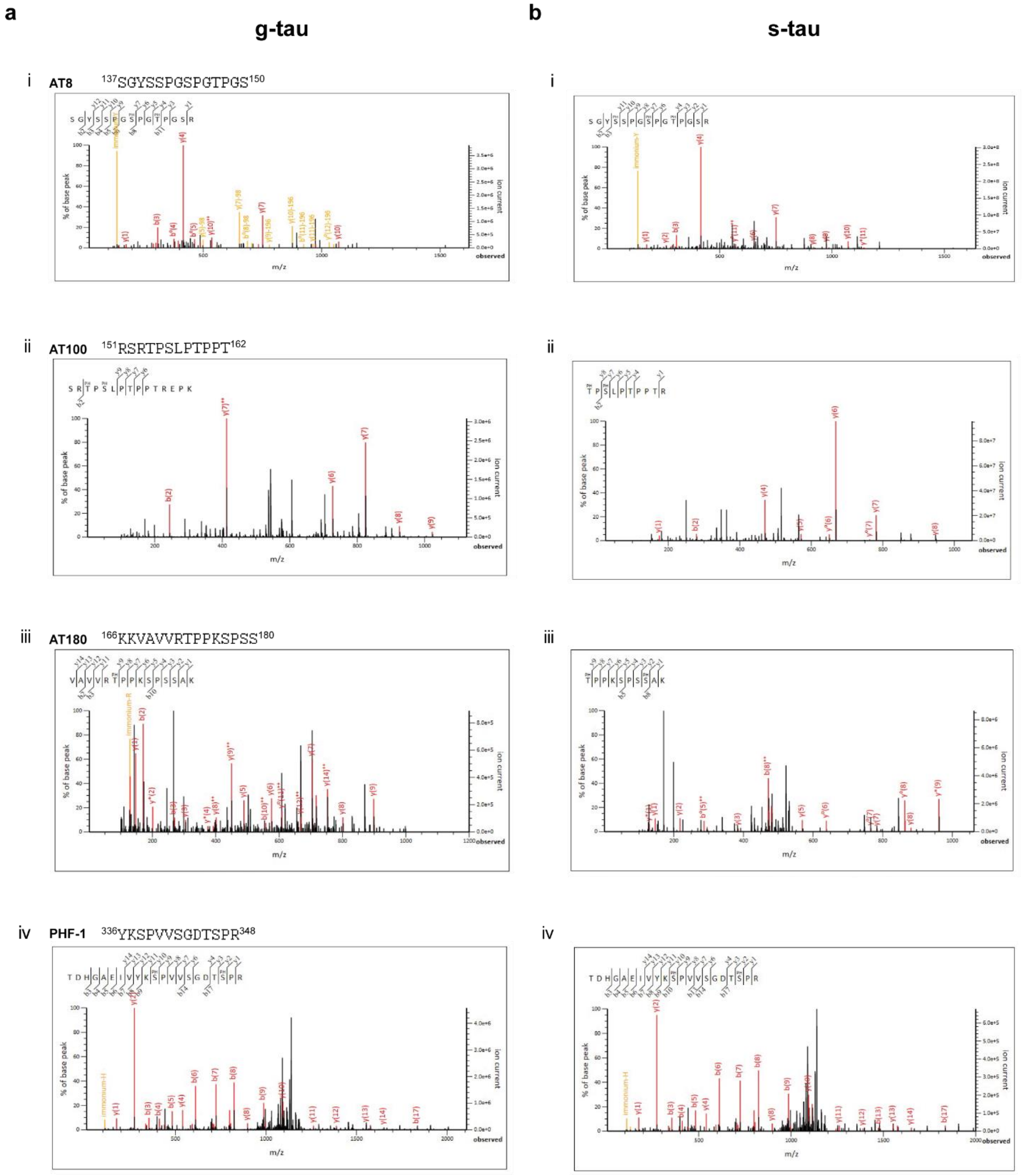

Supplementary Figure 1. Characterization of the phosphorylation state of g-tau and s-tau by mass spectrometry.

Representative LC-MS/MS spectra from three independent experiments revealed both a) g-tau and b) s-tau tau were phosphorylated at AD-specific epitopes, including targeting sites of (i) AT8, (ii) AT100, (iii) AT180, and (iv) PHF1.

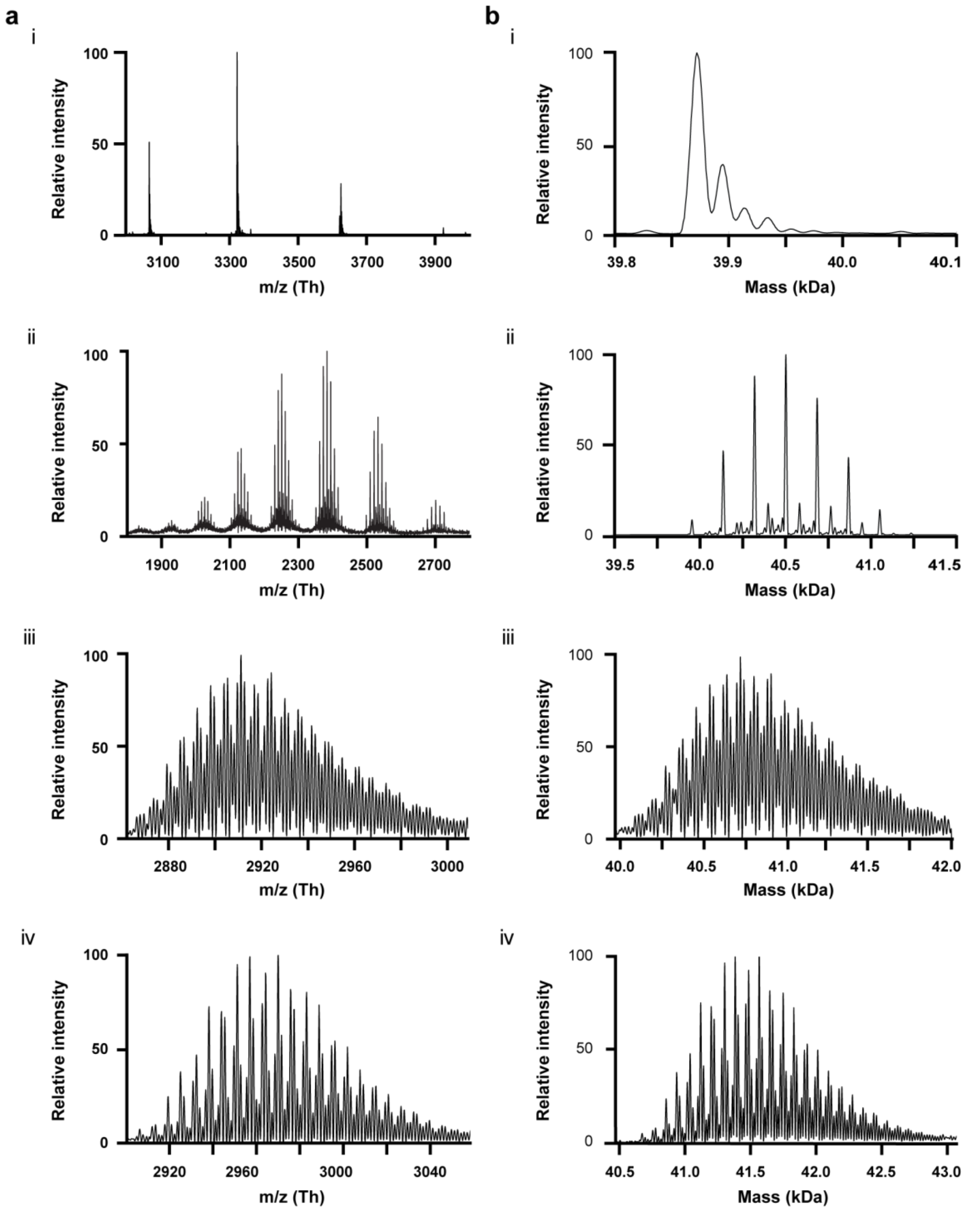

Supplementary Figure 2. Characterization of the phosphorylation state of g-tau and s-tau by mass spectrometry.

a. Representative raw data from three independent high-resolution native mass spectrometry experiments for (i) unmodified WT tau, (ii) pka-tau, (iii) g-tau, and (iv) s-tau.

b. Representative deconvoluted mass spectrometry results from three independent experiments (i) unmodified WT tau, (ii) pka-tau, (iii) g-tau, and (iv) s-tau indicating unmodified WT tau ranged between 39.8 kDa and 40.0 kDa, pka-tau between 40.0 and 41.0 kDa, g-tau between 40.0 and 42.0 kDa while s-tau exhibited a mass distribution between 40.5 and 43.0 kDa.

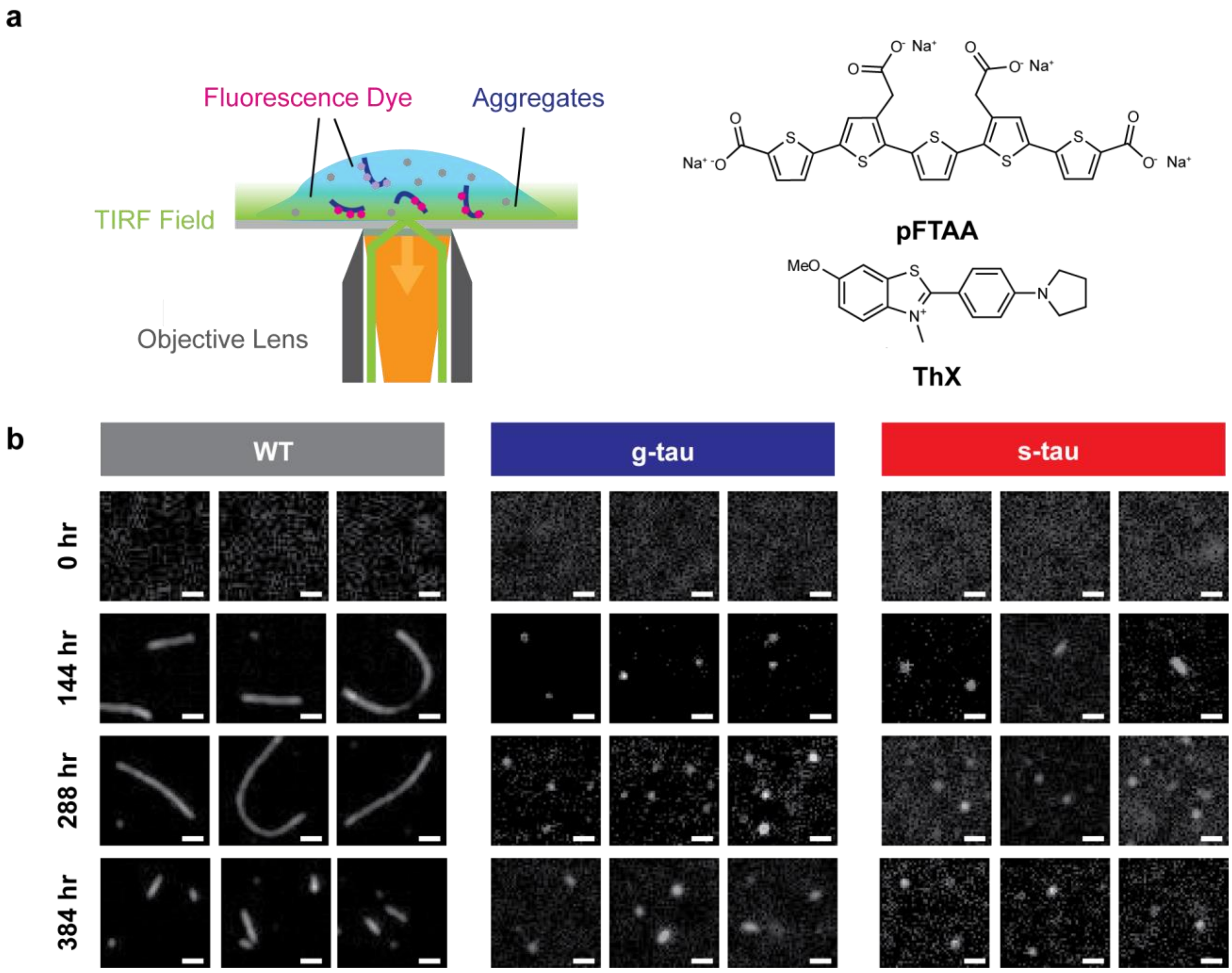

SFigure 3. Morphological characterization of aggregates formed by different tau species using fluorescence microscopy.

a. A pictorial representation of the TIRF microscopy setup workflow along with molecular structures of the fluorescence dyes being used, namely pFTAA and ThX.
b. Representative diffraction-limited images across three independent experiments for each tau species at different timepoints of incubation: WT tau grew into fibrillar aggregates while both g-tau and s-tau formed small non-fibrillar aggregates. Scale bar: 1.0 μm.

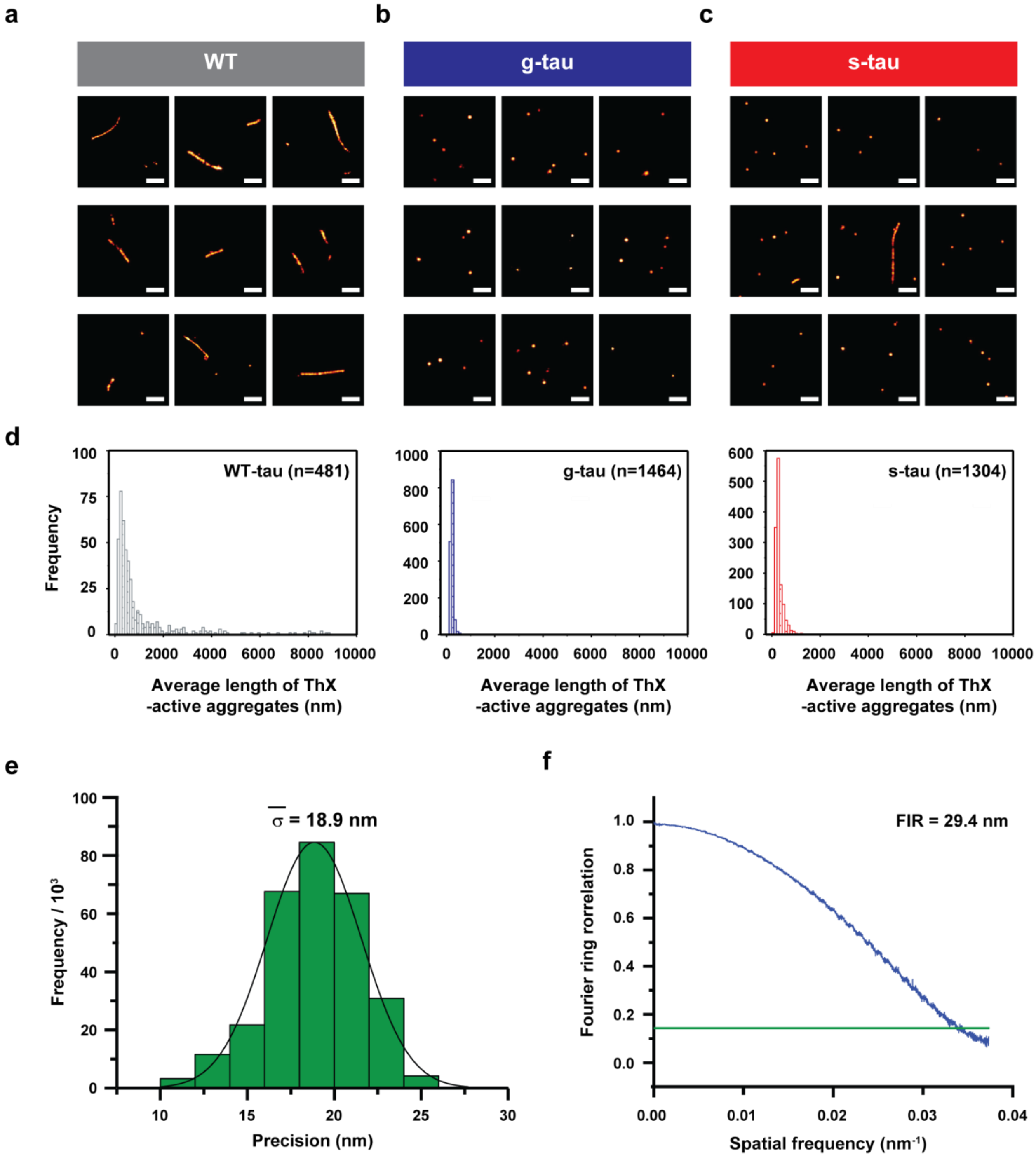

Supplementary Figure 4. Super-resolution imaging of tau aggregates.

a. Representative super-resolved images of WT aggregates after 96 h of incubation. Scale bar: 1.0 μm.
b. Representative super-resolved images of g-tau aggregates after 96 h of incubation. Scale bar: 1.0 μm.
c. Representative super-resolved images of s-tau aggregates after 96 h of incubation. Scale bar: 1.0 μm.

d. Size distribution histograms for different tau aggregates after 96 h of incubation. More than 25% of WT tau aggregated to fibrillar aggregates that were longer than 1000 nm, whereas no g-tau and only 1.2% of s-tau were able to do so. On the other hand, about 99% of g-tau and 89% of s-tau aggregates stayed below a length of 500 nm, while only half of WT tau aggregates were under that threshold.
e. Histogram of localization precision of single ThX blinking events with a mean localization precision of 18.9 nm.
f. Fourier ring correlation analysis showing the Fourier image resolution (FIR) of the ThX super-resolved image of tau aggregates is at 29.4 nm.

For a-c, images were representative across three independent experiments for each tau species.

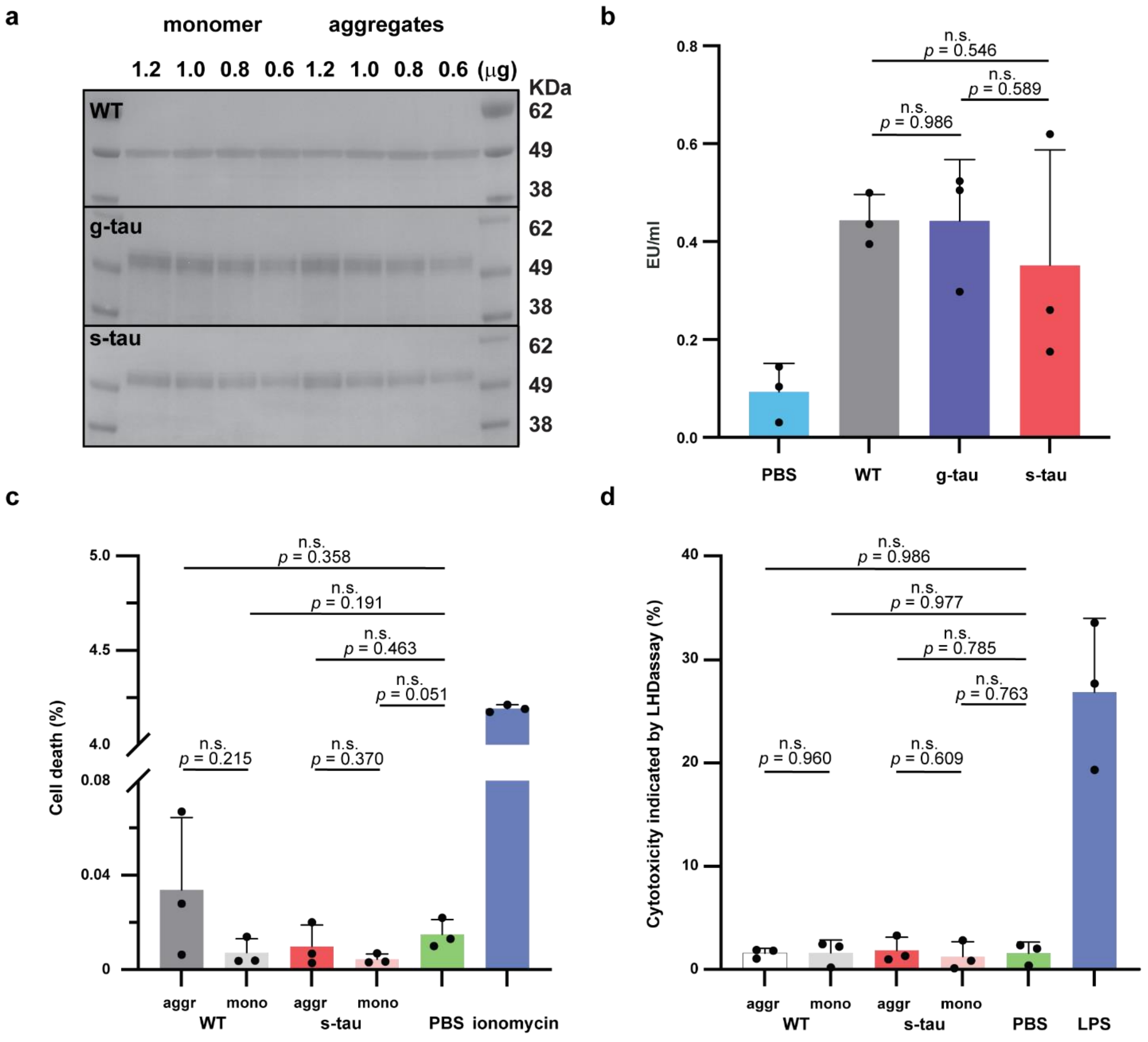

Supplementary Figure 5. Quantification of the degree of aggregation and residua l LPS level for each tau species.

a. Representative SDS-PAGE (4-12%, stained by Coomassie blue) across three independent experiments quantified by densitometry allowed us to estimate the extent of aggregation at 96 h time point. By running various concentrations of tau aggregates and monomer from 0.6 μg to 1.2 μg, we first confirmed the selected concentrations were within the linear range of our densitometric detection. Then by comparing the band intensities between aggregates and monomer, our SDS-PAGE result demonstrated that about 2% of WT tau and 1% of g-tau and s-tau had formed aggregates.
b. To ensure that our results were not confounded by endotoxins contamination of our recombinant tau proteins purified from bacterial cultures, LPS contamination was removed after the phosphorylation reactions by using Pierce™ high-capacity endotoxin removal spin columns. To confirm the efficacy of this removal procedure, the LAL assay was employed to quantify the levels of any remaining LPS. At 2 μM monomer equivalent concentration WT tau contained 0.47 ± 0.05 EU/mL, g-tau, 0.37 ± 0.13 EU/mL, and s-tau 0.43 ± 0.25 EU/mL with PBS buffer control 0.13 ± 0.03 EU/mL, all of which are classified as low levels of LPS contamination (0.5 EU/mL) in cell cultures (Error bars represent ± s.d. of n = 3 for each sample). The *P* values are based on unpaired Student's t test: n.s., non-significant.Error bars in c and e represent ± s.d. of three independent experiments.
c. To ensure such proinflammatory response was directly caused by tau aggregates rather than as a secondary event induced by cell death due to the presence of protein aggregates, Live-or-DyeTM Fixable Viability staining experiment was performed on the THP-1 cells and the results were analyzed by fluorescence activated cell sorting (FACS) technique, indicating that tau aggregates and monomer of both species did not cause significant cellular death as compared to PBS buffer control under our 24 h treatment scheme.
d. To also check if tau aggregates cause any necrotic or pyroptotic event, lactate dehydrogenase (LDH) assay was conducted on THP-1 cells when treated with different tau samples, and our results indicated that tau aggregates and monomer of both species did not significantly compromise membrane integrity as compared to PBS buffer control under our 24 h treatment scheme.

The *P* values are based on unpaired Student's t test: n.s., non-significant. Data are presented as mean values ± s.d. of three independent experiments.

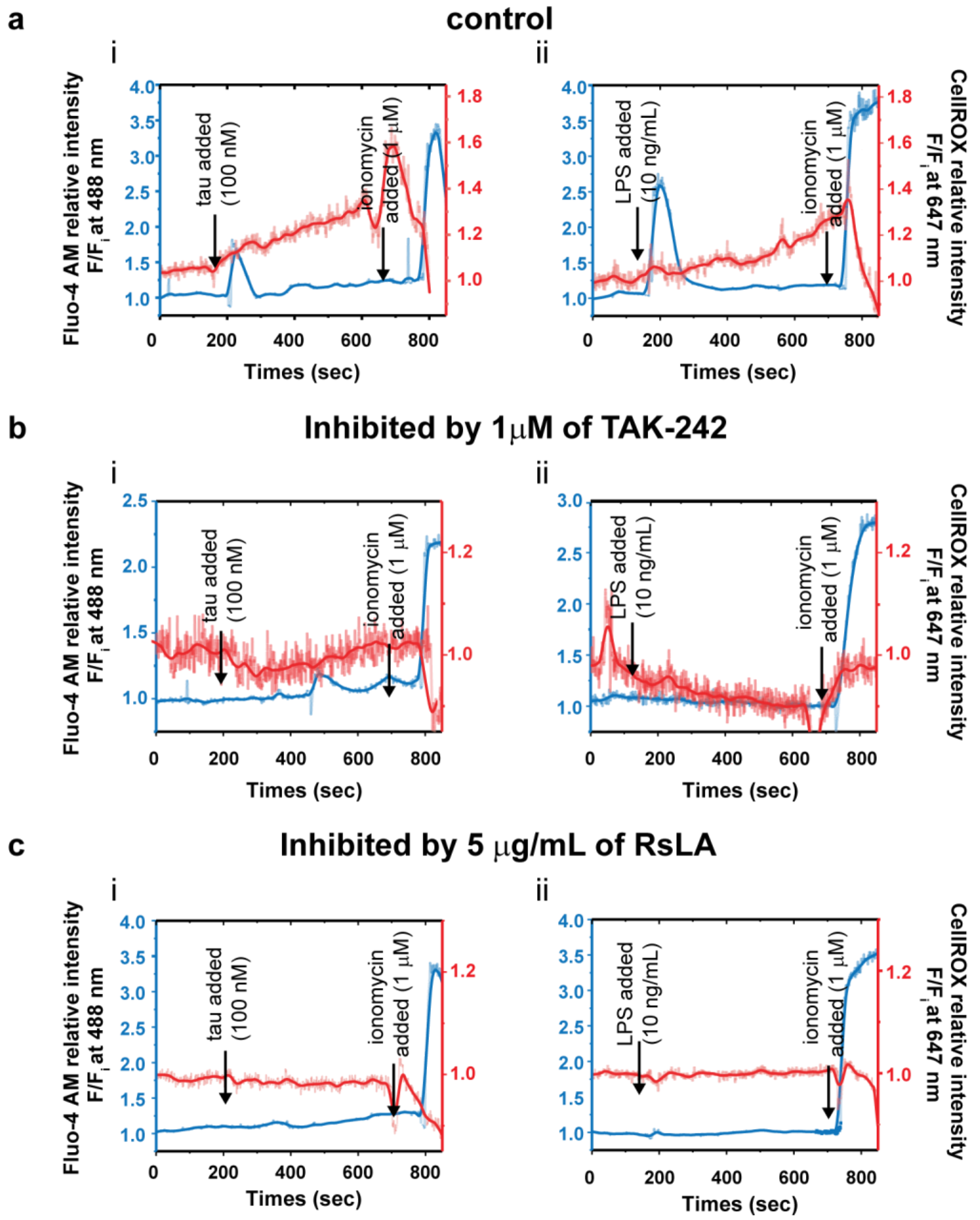

Supplementary Figure 6. TLR4 inhibitors can effectively prevent calcium trans ients and ROS production elicited by s-tau aggregates in human macrophages.

Representative calcium and ROS signal traces after the application of corresponding stimuli, either s-tau aggregates at 100 nM or LPS at 10 ng/mL under different conditions. In comparison with the control condition in a, TAK-242 in b and RsLA c exhibited strong inhibitory effect towards s-tau aggregates and LPS the canonical TLR4 agonist in terms of macrophage activation, suggesting s-tau stimulated calcium transient and ROS production through a TLR4 dependent manner.

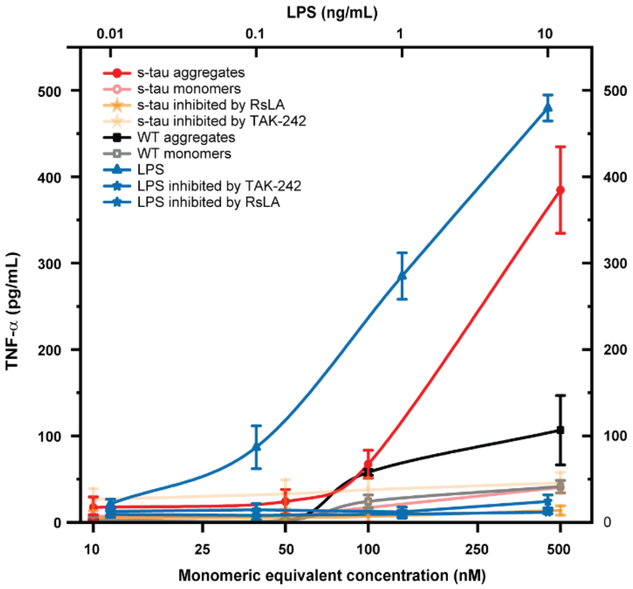

Supplementary Figure. 7 Hyperphosphorylated tau elicited TLR4-mediated in flammation in human macrophages.

As a positive control, various concentrations of LPS (varying from 10 pg/mL to 10 ng/mL) were applied to elicit robust TLR4 activation, resulting in the release of TNF-α in a dose-dependent fashion. Our results also showed s-tau aggregates can cause more potent proinflammatory response than WT aggregates after 24 hours of incubation ($F_{(1,7)} = 67.07$). Since monomer controls did not elicit any significant response in comparison with their corresponding aggregate samples ($F_{(1,7)} = 16.59$ between WT aggregates and monomers, $F_{(1,7)} = 167.46$ between s-tau aggregates monomers), ATP and LPS contamination were controlled. The involvement of TLR4 was subsequently tested: the inhibitory effect of TAK-242 was first being verified since the inhibitor successfully suppressed the level of TNF-α ($F_{(1,7)} = 1120.74$). When inhibited by TAK-242, the s-tau can no longer elicit any significant inflammatory response, thereby suggesting the observed inflammation caused by tau aggregated was mediated by TLR4. The *P* values are based on one-way measures ANOVA: data are presented as mean values ± s.d. of n = 4 independent experiments for each condition and all $p < 0.001$.

Previous reports		Gong et al, J Neural Transm 2005			Liu et al, FEBS letter 2006			Hanger et al, JBC 2007			Tepper et al. JBC 2014		Mair et al, Anal. Chem 2016			current study		
Species		PHF tau	PKA	GSK	PKA	GSK	PKA+GSK	PHF tau	PKA	GSK-beta	P12	P20	deP-tau	P-tau	PP-tau	PKA	PKA + GSK3b	PKA + SAPK4
AA	Site																	
T	17																	yes
Y	18							yes										
Y	29															yes	yes	yes
T	30															yes	yes	yes
T	39	yes														yes	yes	yes
S	46	yes		yes				yes		yes								
T	50			yes				yes		yes								
S/T	68/69	yes						yes				yes		yes	yes			
T	71							yes										
T	76														yes			
T	95																	
T	101																	
T	102																	
T	111																	yes
S	113							yes										yes
T	123	yes						yes										
S	131										yes	yes						
T	135										yes	yes						
S	137	yes																
T	149									yes	yes	yes						
T	153	yes						yes		yes	yes	yes	yes	yes	yes			yes
T	169																	
T	175	yes						yes		yes				yes	yes		yes	yes
T	181	yes		yes			yes	yes		yes			yes	yes	yes		yes	yes
T / T	175 / 181							yes		yes				yes	yes			
S	184			yes				yes						yes	yes			yes
S	185							yes										yes
S	191							yes									yes	yes
S	195			yes													yes	
Y	197							yes									yes	
S	198	yes		yes				yes	yes								yes	yes
S	199	yes		yes		yes	yes	yes						yes	yes		yes	yes
S	202	yes		yes			yes	yes			yes	yes		yes	yes		yes	yes
T	205	yes		yes		yes	yes	yes			yes	yes		yes	yes		yes	yes
**S+T (AT8)**	202 + 205							yes			yes	yes					yes	yes
S	208	yes						yes									yes	yes
S	210	yes						yes	yes	yes				yes	yes		yes	yes
T	212	yes		yes		yes	yes	yes				yes		yes	yes	yes	yes	yes
S	214	yes	yes		yes		yes	yes	yes			yes	yes	yes	yes	yes	yes	yes
T	217	yes		yes			yes	yes		yes			yes	yes	yes		yes	yes
**T + S (AT100)**	212 + 214							yes				yes		yes	yes	yes	yes	yes
T + T	212 + 217														yes			
T	220									yes								
**T (AT180)**	231	yes		yes		yes	yes	yes		yes		yes	yes	yes	yes		yes	yes
S	235	yes		yes				yes		yes	yes	yes		yes	yes		yes	yes
T + S	231 + 235							yes		yes		yes		yes	yes			
S	237	yes						yes		yes								yes
S	238	yes						yes										yes
S	241	yes								yes								
T	245								yes	yes								yes
S	258							yes	yes	yes								
S	262	yes	yes	yes	yes		yes	yes	yes	yes				yes	yes	yes	yes	yes
T	263											yes						
S	285	yes								yes								
S	289							yes		yes							yes	yes
S	293														yes			
S	305	yes							yes	yes		yes				yes	yes	yes
Y	310											yes						
S	316											yes						yes
S	324	yes	yes						yes	yes		yes		yes	yes			
S	341																	yes
S	352	yes							yes	yes								
S	356	yes	yes	yes				yes	yes	yes					yes	yes	yes	yes
T	361																	yes
T	373									yes								
T	386																	
Y	394							yes										yes
S	396	yes		yes		yes	yes	yes		yes		yes		yes	yes		yes	yes
S	400	yes		yes				yes		yes							yes	yes
T	403	yes		yes				yes									yes	yes
S	404	yes		yes		yes	yes	yes				yes		yes	yes		yes	yes
**S+S (PHF-1)**	396 + 404					yes	yes	yes				yes		yes	yes		yes	yes
S	409	yes	yes		yes		yes	yes	yes	yes						yes	yes	yes
S	412	yes						yes	yes							yes	yes	yes
S	413	yes						yes	yes								yes	yes
T	414																yes	yes
S	416	yes	yes					yes								yes	yes	yes
S	422	yes					yes	yes						yes	yes	yes	yes	yes
T	427							yes										
S	433							yes										
S	435							yes	yes									yes

Supplementary Table 1. Comparison with previous reports on AD-derived PHF tau [9] and other (hyper)phosphorylated tau synthesized *in vitro*[13,18–20] illustrating our hyperphosphorylated tau g-tau and s-tau were the most disease-relevant species in terms of their phosphorylation sites.
