# Supplementary material for: Hyperphosphorylated tau self-assembles into amorphous aggregates eliciting TLR4-dependent responses
Source: Nat Commun. 2022 May 16;13:2692. doi: 10.1038/s41467-022-30461-x (PMC9110413; doi:10.1038/s41467-022-30461-x)
Supplement: Supplementary file 2 — Reporting Summary [file 41467_2022_30461_MOESM2_ESM.pdf]

nature research

Corresponding author(s): David Klenerman

Last updated by author(s): Apr 15, 2022

Reporting Summary

Nature Research wishes to improve the reproducibility of the work that we publish. This form provides structure for consistency and transparency in reporting. For further information on Nature Research policies, see our Editorial Policies and the Editorial Policy Checklist.

Statistics

For all statistical analyses, confirm that the following items are present in the figure legend, table legend, main text, or Methods section.

n/a	Confirmed	
☐	☒	The exact sample size (*n*) for each experimental group/condition, given as a discrete number and unit of measurement
☐	☒	A statement on whether measurements were taken from distinct samples or whether the same sample was measured repeatedly
☐	☒	The statistical test(s) used AND whether they are one- or two-sided<br>*Only common tests should be described solely by name; describe more complex techniques in the Methods section.*
☒	☐	A description of all covariates tested
☐	☒	A description of any assumptions or corrections, such as tests of normality and adjustment for multiple comparisons
☐	☒	A full description of the statistical parameters including central tendency (e.g. means) or other basic estimates (e.g. regression coefficient) AND variation (e.g. standard deviation) or associated estimates of uncertainty (e.g. confidence intervals)
☐	☒	For null hypothesis testing, the test statistic (e.g. F, t, r) with confidence intervals, effect sizes, degrees of freedom and P value noted<br>*Give P values as exact values whenever suitable.*
☐	☒	For Bayesian analysis, information on the choice of priors and Markov chain Monte Carlo settings
☒	☐	For hierarchical and complex designs, identification of the appropriate level for tests and full reporting of outcomes
☒	☐	Estimates of effect sizes (e.g. Cohen's *d*, Pearson's *r*), indicating how they were calculated

Our web collection on statistics for biologists contains articles on many of the points above.

Software and code

Policy information about availability of computer code

Data collection	Images acquired using open source Micro-Manager 1.4.
Data analysis	Image analysis was performed using custom scripts programmed in Python 3.5 and MATLABR2019B.

For manuscripts utilizing custom algorithms or software that are central to the research but not yet described in published literature, software must be made available to editors and reviewers. We strongly encourage code deposition in a community repository (e.g. GitHub). See the Nature Research guidelines for submitting code & software for further information.

Data

Policy information about availability of data

All manuscripts must include a data availability statement. This statement should provide the following information, where applicable:

- Accession codes, unique identifiers, or web links for publicly available datasets
- A list of figures that have associated raw data
- A description of any restrictions on data availability

The mass spectrometry proteomics data in this study have been deposited to the ProteomeXchange Consortium via the PRIDE partner repository with the accession code PXD033127[http://www.ebi.ac.uk/pride/archive/projects/PXD033127](Human tau 0N4R isoform hyperphosphorylation LC-MS/MS). The remaining data are available within the Article, Supplementary Information, or Source Data file. Source data are provided with this paper.

Field-specific reporting

Please select the one below that is the best fit for your research. If you are not sure, read the appropriate sections before making your selection.

- [x] Life sciences
- [ ] Behavioural & social sciences
- [ ] Ecological, evolutionary & environmental sciences

For a reference copy of the document with all sections, see nature.com/documents/nr-reporting-summary-flat.pdf

Life sciences study design

All studies must disclose on these points even when the disclosure is negative.

Sample size	Sample size for each experiment was specified in each figure legend, and n=3 was chosen as the minimal replicate number. THP-1 monocytes were seeded at 200,000 cells/mL and 600,000 cells per well and CD14+ primary human macrophages were differentiated with 300,000 cells per well.
Data exclusions	There were no data exclusions in this study.
Replication	The replicate experiments were successful. This forms the basis of the sample sizes described in the figure legends of each experiment.
Randomization	Protein samples, drugs (inhibitors and positive controls), and cells are aliquoted and subsequently selected randomly. Protein samples and cells used for this study were selected randomly and analyzed equally.
Blinding	LC-MS/MS and native mass spec (Fig. 1c&d), the membrane permeability assay (Fig. 3b), and qPCR gene expression study were carried out as blind experimenta, in all other experiments blinding was not possible as the same investigator(s) were simultaneously preparing and running the samples.

Reporting for specific materials, systems and methods

We require information from authors about some types of materials, experimental systems and methods used in many studies. Here, indicate whether each material, system or method listed is relevant to your study. If you are not sure if a list item applies to your research, read the appropriate section before selecting a response.

Materials & experimental systems

n/a	Involved in the study	
x		Antibodies
	x	Eukaryotic cell lines
x		Palaeontology and archaeology
x		Animals and other organisms
	x	Human research participants
x		Clinical data
x		Dual use research of concern

Methods

n/a	Involved in the study	
x		ChIP-seq
x		Flow cytometry
x		MRI-based neuroimaging

Eukaryotic cell lines

Policy information about cell lines

Cell line source(s)	The THP-1 cells were obtained from ATCC-TIB-202 from LGC Ltd, Middlesex, UK.
Authentication	No additional authentication of this cell line was performed.
Mycoplasma contamination	Cells were tested negative for mycoplasma contamination.
Commonly misidentified lines (See ICLAC register)	No commonly misidentified cell lines were used.

Human research participants

Policy information about studies involving human research participants

Population characteristics	The human primary monocytes were extracted from blood from donors who gave their samples voluntarily without any compensation. They gave informed consent via a Good Clinical Practice registered member of staff from Royal Papworth Hospital NHS Foundation Trust Cambridge under local ethics committee approval (REC No. 12/WA/0148). No human derived cells were stored after the experiments had been completed.
Recruitment	Age between 30 and 40, male and female, no genotype information required, healthy volunteers with no current illness on medication, and no history of chronic disorders. The volunteers are not patients, so selection is not made on genotype, disease or medication. The donors are also part of the National Blood and Transplant regular blood donation scheme and are therefore regularly screened for blood borne diseases. The selection is random over age, sex, and other relevant categories.
Ethics oversight	Royal Papworth Hospital NHS Foundation Trust Cambridge local ethics committee approval (REC No. 12/WA/0148)

Note that full information on the approval of the study protocol must also be provided in the manuscript.
